# Detection of VOCs and Biogenic Amines Through Luminescent Zn–Salen Complex-Tethered Pyrenyl Arms

**DOI:** 10.3390/molecules29235796

**Published:** 2024-12-08

**Authors:** Roberta Puglisi, Caterina Testa, Sara Scuderi, Valentina Greco, Giuseppe Trusso Sfrazzetto, Manuel Petroselli, Andrea Pappalardo

**Affiliations:** 1 Department of Chemical Science, University of Catania, 95125 Catania, Italy; roberta.puglisi@unict.it (R.P.); caterina.testa@phd.unict.it (C.T.); sarascuderi@icloud.com (S.S.); valentina.greco@unict.it (V.G.); giuseppe.trusso@unict.it (G.T.S.); 2 Center for Supramolecular Chemistry and Catalysis and Department of Chemistry, College of Science, Shanghai University, Shanghai 200444, China; mpetroselli@iciq.es

**Keywords:** VOC, biogenic amines, Salen complexes, fluorescence, DFT, supramolecular chemistry

## Abstract

Amines are produced through various industrial and biological processes, contributing significantly to atmospheric pollution, particularly in the troposphere. Moreover, amine-related pollution raises global concerns due to its detrimental effects on human health, environmental quality, and the preservation of animal species. Low-molecular-weight volatile amines, categorized as volatile organic compounds (VOCs), are present in the atmosphere, and they represent the main cause of air pollution. Biogenic amines, resulting from the natural decarboxylation of amino acids, are released into the environment from both natural and industrial sources. Several methods have been developed so far to detect amines in the environment. In this study, we present a novel fluorescent receptor based on a Zn–Salen complex, functionalized with pyrenyl moieties and a chiral diamine bridge, to enhance its affinity for a broad range of amines. Fluorescence titrations and density functional theory (DFT) calculations reveal and explain the high binding affinity of this receptor toward selected amines, demonstrating its potential as an effective tool for amine detection.

## 1. Introduction

The increasing rate of industrialization worldwide is leading to significant health and environmental issues that need to be addressed. Growing attention is paid to the production and release of amines into the environment due to their potential toxicity for humans, animals, and microorganisms. Amines are widespread compounds, derived from both natural and industrial sources [1], with hundreds of important derivatives. The anthropogenic use of amines and amine-related compounds, combined with the risks posed by their release into the environment, highlights the importance of developing efficient real-time detection systems to monitor amine levels, particularly below hazardous concentrations [2,3].

Volatile amines and their derivatives are potentially toxic when present in the atmosphere and in the human body. The most common and abundant amines found in the air are low-molecular-weight aliphatic amines with a 1–6 carbon chain, namely ethylamine, propylamine, butylamine, and hexylamine [4]. These highly volatile derivatives belong to the broader category of volatile organic compounds (VOCs), and they are major contributors to air pollution [5]. In addition to their unpleasant smell, exposure through inhalation or skin contact with these compounds can result in serious health risks, such as eye and skin irritation, conjunctivitis, and corneal edema. Thus, monitoring these compounds is critical for preventing toxicological effects. The main sources of low-molecular-weight amines include several anthropogenic processes, such as animal husbandry, beverage and food processing, agrochemical production, and pharmaceutical manufacturing.

Additionally, amines can be naturally produced as by-products of the normal metabolic processes of microorganisms, plants, and animals, primarily through the decarboxylation of neutral amino acids [6]. This process is the most common way for amine synthesis to occur in foods, and the resulting aromatic amines, named “biogenic”, can make food toxic [7]. Indeed, high levels of biogenic amines can be found in spoiled food [8]. Moreover, biogenic amines show thermostability, which makes them resistant to the thermal treatments used in food processing.

Also, biologically active amines such as tryptamine, β-phenylethylamine, and tyramine have significant physiological effects in humans, acting as psychoactive or vasoactive agents, by influencing neurotransmitters and the vascular system. They are found in trace amounts in various foods, particularly those that are fermented, such as cheese, sausage, and red wine. Moreover, structural similarities with catecholamines have led to the development of synthetic analogs, illegally synthesized as psychotropic substances, such as amphetamines [9]. In particular, β-phenylethylamine and tryptamine have gained interest as potential “endogenous amphetamines”, finding application as possible biomarkers and etiologic factors for psychiatric disorders [10].

It is worth mentioning that, among the wide variety of amine compounds, chiral amines are present in numerous natural products and biologically active compounds, and they play an important role in the synthesis of various drugs as fundamental intermediates [11].

In light of the above-discussed significant role that amine-based compounds play in synthetic, biological, and physiological processes, the monitoring of these amines’ levels in several environments is a fundamental goal [12]. Nowadays, traditional methods for detecting amines include high-performance liquid chromatography (HPLC) [13,14], gas chromatography–mass spectrometry (GC-MS) [15], and cyclic voltammetry [16]. Despite the excellent and reliable results obtained using these instrumental techniques, they require expert personnel and time-consuming sample preparation. For these reasons, a detection method that is sensitive, fast, inexpensive, and available to non-specialized operators would be desirable. In this context, fluorescence- and colorimetric-based molecular sensors are gaining considerable attention. These sensors offer numerous advantages, such as low-cost starting materials, simple detection systems, high sensitivity, the portability of the equipment, quick response times, and ease of use [17,18,19,20,21,22,23]. Molecular-based fluorescent sensors are optically active molecules (receptors) able to provide an easy, measurable, or visible response upon interaction with the target analyte.

Typically, fluorescent receptors capable of interacting covalently with amines give rise to a measurable variation in their emission properties [24]. This type of recognition presents some disadvantages, such as poor selectivity and the possibility of false positive responses, due to the presence of competitive analytes.

In contrast, the less explored supramolecular approach offers more specific recognition due to the complementarity between the receptor (host) and the target analyte (guest), exploiting non-covalent interactions such as hydrogen bond, metal coordination, CH-π and/or π-π interactions, as well as Lewis acid–base interactions. Moreover, affinity and selectivity can be increased through the combination of multiple interaction sites between host and guest. Finally, these weak interactions lead to a reversible recognition process, paving the way for reusable sensors.

Despite the advantages of this approach, only a few fluorescent supramolecular receptors able to detect amines and their derivatives have been reported, including macrocycles [25,26,27], metal–organic complexes [28,29,30,31,32], and bodipy fluorescent scaffolds [33]. Among others, metal–Salen complexes have emerged as particularly effective fluorescent receptors for a wide range of analytes [34]. This class of receptor displays the perfect match between detection efficiency, ease of synthesis, versatile functionalization, and fluorescent response. The metal center, acting as an acidic Lewis site, can interact with various Lewis base species, and the functionalization of the Salen backbone can increase the number of interaction sites, resulting in more efficient multi-topic recognition [35]. Notably, Zn–Salen complexes have shown excellent abilities as receptors for amine detection [36,37]. The sensing mechanism relies on the Lewis acid–base interaction between the Zn(II) ion, acting as the acidic site, and the amino group of various amines, acting as the basic site. This interaction results in the axial coordination of amine to the Zn(II) ion, leading to a square pyramidal geometry.

**Figure 1:**
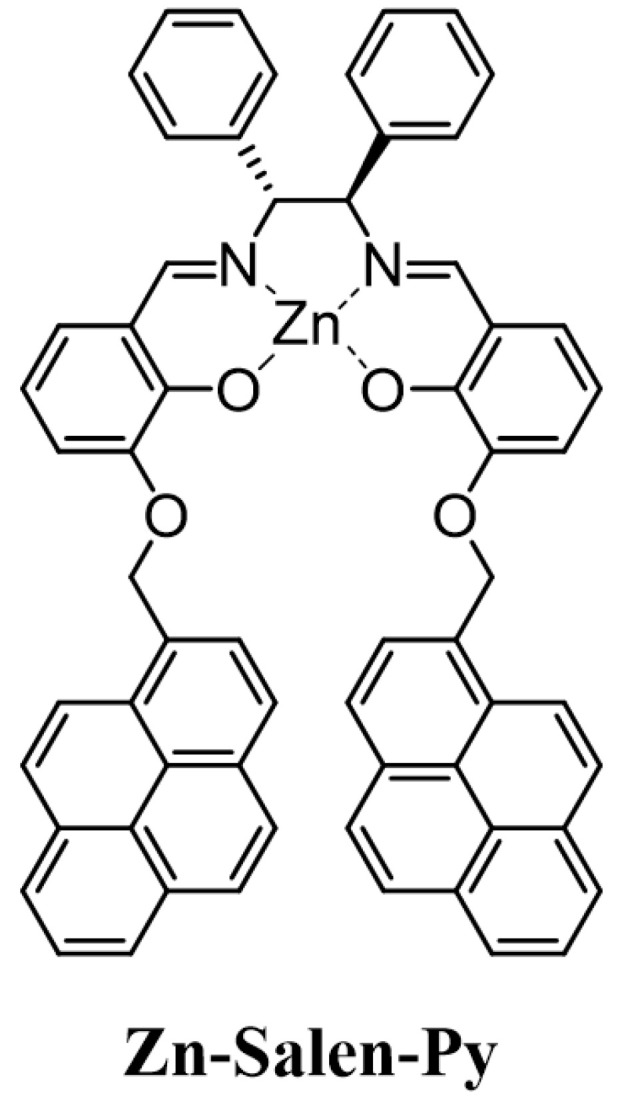
Structure of the Zn–Salen–Py complex.

**Figure 2:**
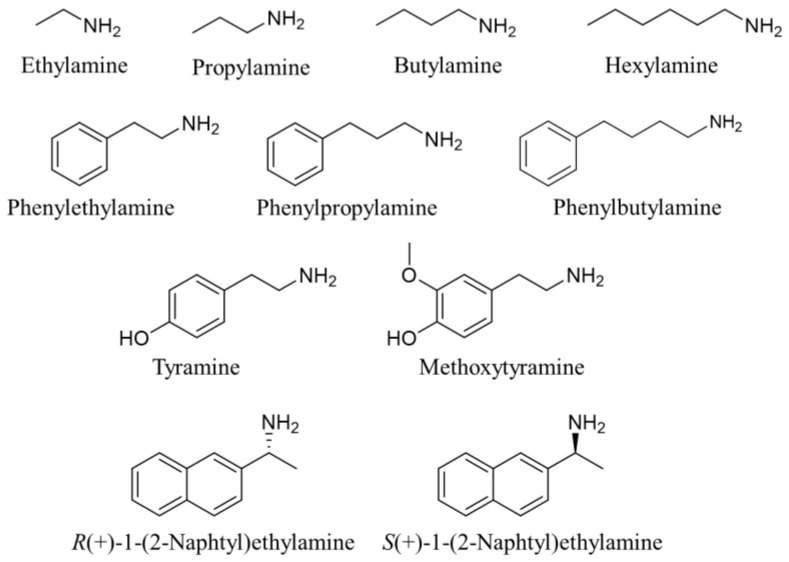
Structures of relevant amines identified as VOCs (ethylamine, propylamine, butylamine, and hexylamine); biogenic amines (phenylethylamine, phenylpropylamine, phenylbutylamine, tyramine, methoxytyramine; important chiral intermediates: *R*(+)-1-(2-Naphtyl)ethylamine and *S*(−)-1-(2-Naphtyl)ethylamine.

Herein, we present the design and synthesis of a novel fluorescent Zn–Salen receptor (Zn–Salen–Py), bearing a chiral (1*R*,2*R*)-diphenylethylenediamine bridge and two pyrenyl arms in the salicylidene moiety (Figure 1), for the detection of properly selected aliphatic and aromatic amines (Figure 2). In particular, the introduction of two pyrenyl arms to the Salen scaffold should promote CH-π interactions with the aliphatic chains of amines, as well as additional π-π interactions with the aromatic amines. Moreover, the presence of the chiral bridge in the diamine backbone can potentially drive enantioselective discrimination between chiral amines.

The recognition properties of Zn–Salen–Py towards selected aliphatic, aromatic, and chiral amines were investigated through fluorescence titrations, monitoring the changes in the emission profile upon the progressive addition of each amine. Interestingly, a defined trend in the binding affinity was observed, and the remarkable enantioselectivity found with chiral amines was also assessed through DFT calculations.

## 2. Results and Discussion

**Scheme 1:**
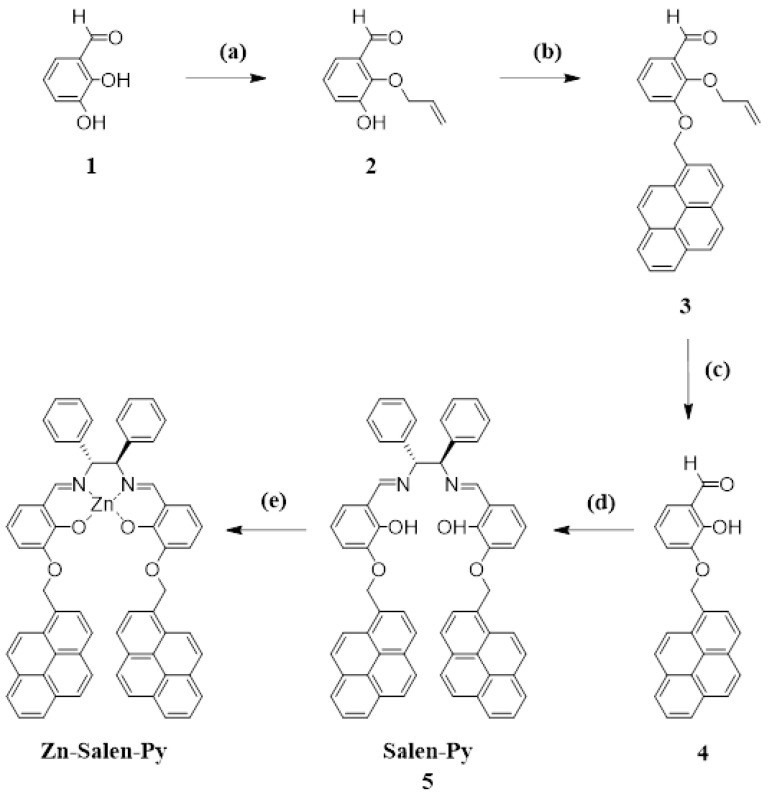
Reagents and conditions: (**a**) allyl bromide, K_2_CO_3_, CH_3_CN, reflux, 18 h, 64%; (**b**) 1-(bromomethyl)pyrene, K_2_CO_3_, CH_3_CN, reflux, 24 h, 46%; (**c**) Pd(OAc)_2_, PPh_3_, Et_3_N, HCOOH, 80% EtOH, reflux, 45 min, 75%; (**d**) (1*R*,2*R*)-(+)-1,2-diphenylethylenediamine, EtOH, reflux, 15 h, 88%; (**e**) Zn(CH_2_CH_3_)_2_, toluene, r.t., 24 h, 98%.

The synthesis of Zn–Salen–Py was accomplished starting from 2,3-dihydroxybenzaldehyde (**1**), and it was initially protected in position 2 with allyl bromide to yield 2-allyloxy-3-hydroxybenzaldehyde **2** (Scheme 1) [38,39]. Afterwards, pyrene moiety was introduced through the reaction with 1-(bromomethyl)pyrene to obtain derivative **3**. The removal of the allyl group through the Tsuji–Trost reaction afforded compound **4**, which was reacted with (1*R*,2*R*)-(+)-1,2-diphenylethylenediamine to obtain the Salen–Py ligand. The final step provided the introduction of the Zn metal ion using diethyl–Zinc, thus obtaining the desired Zn–Salen–Py receptor, which was characterized via ^1^H and ^13^C NMR spectroscopy and HRMS (see the Supplementary Materials Figures S1–S11).

It is known from the literature that Zn–Salen complexes are likely to form dimers or higher-order aggregates in non-coordinating solvents such as toluene or chloroform [40,41,42]. This phenomenon is due to the tetra-coordination of the zinc ion through the nitrogen and oxygen donor atoms of the Salen ligand, leaving the fifth axial coordination site available for interactions with a Lewis base guest. As a result, solvents with Lewis base sites, such as methanol, which has oxygen lone pairs, can complete the coordination sphere of the zinc metal ion, stabilizing the complex in its monomeric form. To avoid such aggregation phenomena, the optical properties of the receptor were investigated in methanol, as a coordinating solvent, via UV-Vis and fluorescence spectroscopies. Notably, an interesting absorption profile was observed for a Zn–Salen–Py solution (1 × 10^−5^ M) in methanol. The solution shows a band at 347 nm with two shoulders at 330 and 318 nm, attributed to the three vibronic bands of the S_n_ ← S_0_ (n = 0, 1, 2) pyrene transitions, and another band with two components at 275 and 265 nm ascribed to the π–π* transition of the aromatic part and n–π* electronic transitions of the nonbonding electrons of the azomethine nitrogen atoms (see Supplementary Materials Figures S12 and S13) [43]. Fluorescence spectra of the Zn–Salen–Py solution (1 × 10^−5^ M) in methanol, upon excitation with 330 nm or 350 nm radiation, were recorded (see Supplementary Materials Figure S14). In both excitation wavelengths, two strong emissions were observed for the solution, 390 and 470 nm when λ_ex_= 330 nm, and 400 and 491 nm when λ_ex_= 350 nm. In particular, the emission at 390–400 nm was ascribed to the monomeric fluorescence of the pyrenyl substituents, while the band at 470–490 nm is typical of the formation of two types of excimers, as demonstrated by a low energy asymmetry [44,45,46]. Furthermore, emission bands above 500 nm were ascribed to the Salen moiety contribution [47]. The significant fluorescence properties of the Zn–Salen–Py system and the large Stokes shift, ranging from 40 to 140 nm, suggest its potential for amines’ detection.

Hence, fluorescence titrations were conducted using a 1 × 10^−5^ M solution of Zn–Salen–Py in methanol to investigate the recognition properties of Zn–Salen–Py towards relevant amine guests, including ethylamine, propylamine, butylamine, and hexylamine as aliphatic amines (see Supplementary Materials Figures S15–S18), given their role as VOCs in the atmosphere and their significant impact on human health. Moreover, recognition experiments were performed using aromatic amines, such as phenylethylamine, phenylpropylamine, phenylbutylamine, tyramine, and methoxytyramine (see Supplementary Materials Figures S19–S23), due to the great relevance in the biological field, as well as the negative impact on environment quality. Finally, the enantiodiscrimination ability of Zn–Salen–Py was assessed with the chiral amines *R*-(+)-1-(2-Naphthyl)ethylamine and *S*-(+)-1-(2-Naphthyl)ethylamine (see Supplementary Materials Figures S24 and S25).

**Figure 3:**
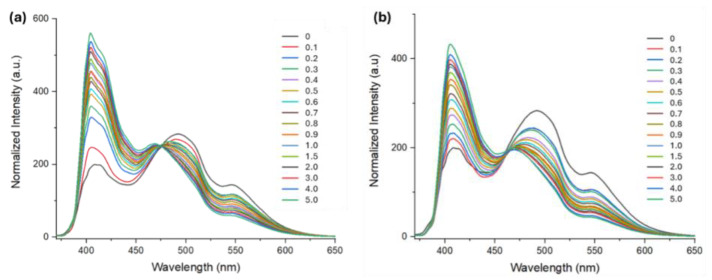
Representative fluorescence titrations of Zn–Salen–Py (1 × 10^−5^ M, λ_ex_= 350 nm) upon addition of (**a**) aliphatic butylamine and (**b**) aromatic phenylbutylamine in the range 0–5 equivalents.

The emission spectra of the Zn–Salen–Py in methanol (1 × 10^−5^ M, λ_ex_= 350 nm) show a similar profile upon the addition of both aliphatic and aromatic amines. Notably, a remarkable turn-on of the emission intensity at 400 nm and a slight decrease in the intensity at 491 nm can be observed when increasing amounts of guests are added. Moreover, for all the selected guests, the band at 491 nm undergoes a blue shift with an increasing amine concentration (Figure 3). This emission behavior, supported by similar findings in the literature, suggests the formation of a host–guest complex driven by a Lewis acid–base interaction between the Zn(II) ion of the receptor, acting as the acidic site, and the amino group of the guests, acting as the basic site. The side chains of both aliphatic and aromatic amines are likely to play a role in enhancing monomeric pyrene emission by interfering with the spatial proximity of the pyrene groups, thus leading to the observed blue shift and the reduction in the intensity of the excimer emission band at 491 nm [48].

Pertinent binding constants (log*K*) of the supramolecular complexes between Zn–Salen–Py and the selected amines were calculated using HypSpec software. The results reported in Table 1 clearly show the high affinity of the Zn–Salen–Py receptor to the selected amines. DFT calculations highlighted a strong Lewis acid–base interaction for all the complexes, accompanied by an NH-π interaction as a secondary interaction (see the Supplementary Materials Figures S26–S32). These results point out the relatively high complexation energy (E_complex_) towards the Zn–Salen–Py host theoretically calculated in the gas phase at B3LYP/631G(d,p) level of theory (Table 2), in agreement with the high affinity measured in MeOH. Interestingly, with both aliphatic (ethyl-, propyl-, butyl-, and hexylamine) and aromatic amines (phenylethyl-, phenylpropyl-, and phenylbutylamine), a gradual increase in the affinity binding of Zn–Salen–Py is observed for more lipophilic amines, as shown in Table 1. For instance, phenylethylamine exhibits a log*K* value of 6.39 ± 0.02, which is slightly lower than the log*K* values of 6.45 ± 0.01 and 6.49 ± 0.01 found for phenylpropylamine and phenylbutylamine, respectively. Notably, this affinity can be primarily attributed to the solvophobic effect in MeOH, with a minor contribution arising from additional aliphatic CH-π interactions. This hypothesis is supported by DFT studies that show similar E_complex_ values in the gas phase for phenylethylamine and phenylbutylamine, considered model aromatic amines (vide infra). In particular, phenylethylamine adopts a single conformation when complexed to a Zn–Salen–Py host, where aromatic CH-π and aliphatic CH–oxygen interactions were detected as secondary stabilizing forces in the formation of the host–guest complex (Figure S27). The E_complex_ value calculated for the phenylethylamine@Zn-Salen-Py complex is 16.2 kcal/mol (Table 2, Entry 3). Similarly, phenylbutylamine shows an E_complex_ value of 15.8 kcal/mol (Table 2, Entry 4), derived from the average of three quasi-isoenergetic conformations and stabilized by similar (or even the same) aromatic CH-π and aliphatic CH-oxygen interactions (Figure S28), as observed in phenylethylamine@Zn-Salen-Py complex. These data are in agreement with the comparable log*K* values found for phenylethylamine and phenylbutylamine, 6.39 ± 0.02 and 6.49 ± 0.01, respectively. The lower E_complex_ value theoretically found for phenylbutylamine must be attributed to the underestimation of the solvophobic effect in our calculations (study in the gas phase). Unfortunately, the computational study of the solvophobic effect in polar and apolar solvents is still quite challenging and unreliable [49,50,51], making its prediction and quantification in supramolecular systems quite hard.

Similarly, when aliphatic amines are considered, the solvophobic effect plays an important role in the formation of the host–guest complex. Ethylamine shows a log*K* of 5.34 ± 0.01, which is slightly lower compared to the log*K* values of 6.03 ± 0.01, 6.21 ± 0.01, and 6.45 ± 0.02 measured for propylamine, butylamine, and hexylamine, respectively. Even in this case, the Lewis acid–base interaction, the solvophobic effect, and CH-π interactions are the driving forces of the host–guest complexation, as previously described for phenylethylamine and phenylbutylamine. The formation of aliphatic CH-π and CH-oxygen interactions is confirmed in DFT studies (Figures S29 and S30) [52], and it contributes to the stabilization of the host–guest complex, as underlined by the high E_complex_ value of 19.2 and 17.9 kcal/mol for ethylamine and hexylamine, respectively (Table 2, entry 1 and 2). Once again, the inability to properly quantify the solvophobic effect through DFT studies leads to underestimating the E_complex_ related to hexylamine, which is the most lipophilic aliphatic amine.

Indeed, the sensitivity of Zn–Salen–Py to selected amines was evaluated through the calculation of the limit of detection (LOD). The results obtained are promising, falling within a range of 0.12–0.35 µM, which is below the hazardous concentration of these amines for humans and animals [3].

**Figure 4:**
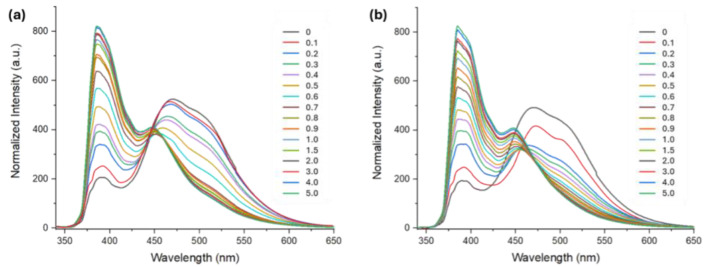
Fluorescence titrations of Zn–Salen–Py (1 × 10^−5^ M, λ_ex_ = 330 nm) in methanol upon the addition of (**a**) *R*-(+)-1-(2-Naphtyl)ethylamine and (**b**) *S*-(+)-1-(2-Naphtyl)ethylamine in range 0–5 equivalents.

As mentioned, the presence of a chiral diamine bridge in the Salen backbone of Zn–Salen–Py prompted us to investigate its enantioselective recognition capabilities in solution. To this end, fluorescence titrations were carried out with the two enantiomers *R*-(+)-1-(2-Naphtyl)ethylamine and *S*-(−)-1-(2-Naphthyl)ethylamine (Figure 4).

Remarkably, strong enantiodiscrimination between the two enantiomers was observed, with log*K* values of 5.61 ± 0.02 for the (*S*)-enantiomer and 7.59 ± 0.07 for the (*R*)-enantiomer, thus leading to an enantiomeric excess (*e*.*e*. = *K*_R_/*K*_S_) of 95. This high selectivity was confirmed in DFT studies on the host–guest complexes formed with chiral amines [53,54]. *R*-(+)-1-(2-Naphtyl)ethylamine shows a single conformer when it interacts with the Zn–Salen–Py host (Boltzmann distribution > 99.9%); multiple aromatic CH-π interactions are observed between the aromatic C-H bond of the guest and the pyrenyl arms of the host (Figure S31). These interactions, along with the Lewis acid–base interaction, lead to a E_complex_ of 14.9 kcal/mol (Table 2, Entry 5). A conformational study on the host–guest complex with the *S*-(−)-1-(2-Naphtyl)ethylamine unveiled, instead, the presence of three quasi-isoenergetic conformations; the most stable (ΔE = −0.91 and −1.76 kcal/mol compared to other two) shows a Boltzmann distribution of 79% (Figure S32). In contrast with the *R*-enantiomer complex, the *S*-(−)-1-(2-Naphtyl)ethylamine@Zn-Salen-Py complex is mainly stabilized via aliphatic CH-π interactions (Figure S32). Aromatic CH-π interactions are instead observed in conformers 2 and 3 (Boltzmann distribution of 79% and 17%, respectively), where the naphthyl group of the guest interacts with both the host’s pyrenyl moieties and phenyl groups (Figure S32). Taking into account the Boltzmann distribution of each conformer, we calculated an averaged E_complex_ value of 10.9 kcal/mol (Table 2, Entry 6), which is 4.0 kcal/mol lower compared to the value found for the *R*-(+)-1-(2-Naphtyl)ethylamine. This result is in accordance with the experimental data, and it justifies the higher enantioselectivity observed for the *R*-(+)-1-(2-Naphtyl)ethylamine enantiomer.

## 3. Materials and Methods

General experimental methods. The NMR experiments were carried out at 27 °C using a Varian UNITY Inova 500 MHz spectrometer (^1^H at 499.88 MHz, ^13^C-NMR at 125.7 MHz) equipped with a pulse-field gradient module (Z axis) and a tunable 5 mm Varian inverse-detection probe (ID-PFG). Photophysical studies were performed in air-equilibrated solutions at room temperature in quartz cuvettes (1.0 cm path length). The UV/Vis absorption spectra were registered using a Jasco V-750 spectrophotometer. Luminescence measurements were carried out with a Jasco FP-8550 spectrofluorometer. The emission was recorded at 90° with respect to the exciting line beam using 5:5 slit-widths for all measurements. Light source: Xe lamp with shielded lamp house, 150 watts; power: 100–240 V, 50/60 Hz, and 350 W. The HRMS experiments were performed using the LTQ XL mass spectrometer, equipped with H-ESI II source, (ThermoFisher, San Jose, CA, USA) in full scan mode from m/z 150 to 2000. All measurements were performed in positive mode with a spray voltage of 3–3.5 kV. The capillary temperature was set to 250 °C, the capillary voltage to 20 V, and the tube lens to 120 V. External calibration was performed using the Pierce LTQ ESI Positive Ion Calibration Solution (ThermoFisher). Data proceeding was performed using the FreeStyle Software, ver. 1.6 SP1 (Thermo Fisher Scientific). All chemicals were reagent-grade and were used without further purification.

Procedure for fluorescence titrations. Two stock solutions of the appropriate receptor and guest (1.0 × 10^−3^ M) in methanol were prepared. From these, different solutions with different receptor/guest ratios were prepared as reported below, and emission spectra, normalized to eliminate the dilution effect, were recorded. Fluorescence titrations with Zn–Salen–Py were carried out using λ_ex_ = 330 nm and 350 nm at 25 °C. With this data treatment, the binding affinities of receptors with selected guests were estimated using HypSpec (version 1.1.33), software designed to extract equilibrium constants from potentiometric and/or spectrophotometric titration data. HypSpec starts with an assumed complex formation scheme and uses a least-squares approach to derive the spectra of the complexes and the stability constants.

Computational detail. Ab initio and density functional calculations were performed using the Gaussian09 program package. 1. The optimization of all involved systems was performed at the B3LYP/6-31G(d,p) level of theory. All structures were subjected to a full conformational search to ensure the reporting of the absolute minimum or the main conformers in accordance with the Boltzmann distribution. Frequencies were calculated and checked to make sure that all of them were positive and that no imaginary frequencies were present. Gaussview software was used as a graphic interface to visualize the molecular structures. Zero-point energy (ZPE) was included in each result.

Synthesis of 2-allyloxy-3-hydroxy benzaldehyde (2): In a 100 mL round-bottom flask, a stirred mixture of anhydrous K_2_CO_3_ (0.349 g, 2.524 mmol), 2,3-dihydroxybenzaldehyde 1 (0.703 g, 5.088 mmol), and allyl bromide (0.22 mL, 2.542 mmol, d = 1.398 g/mL) in dry MeCN (30 mL) was refluxed for 18 h under N_2_. After the evaporation of the solvent, the residue was partitioned between 0.1 M HCl and CH_2_Cl_2_. The organic layer was dried over anhydrous Na_2_SO_4_ and concentrated. The oily residue was purified through column chromatography (SiO_2_, eluent hexane/AcOEt 8:1, v/v). The 2-allyloxy-3-hydroxy benzaldehyde was further purified via crystallization using a mixture of hexane and CH_2_Cl_2_ to give compound **2** (0.290 g, 64%), as a white solid. ^1^H-NMR (500 MHz, CDCl_3_) δ 4.59 (d, *J* = 6.5 Hz, 2 H), 5.35 (d, *J* = 10.0 Hz, 1 H), 5.43 (dd, *J* = 17.0, 1.0 Hz, 1 H), 5.74 (s, 1 H), 6.11 (m, 1 H), 7.15 (t, *J* = 8.0 Hz, 1 H), 7.23 (dd, *J* = 8.0, 1.5 Hz, 1 H), 7.38 (dd, *J* = 7.5, 1.5 Hz, 1 H), 10.27 (s, 1 H) ppm.

Synthesis of 2-allyloxy-3-(pyrenyl-1-methoxy) benzaldehyde (3): In a 100 mL round-bottom flask, a stirred mixture of 2-allyloxy-3-hydroxy benzaldehyde 2 (0.260 g, 1.461 mmol), 1-(bromomethyl)pyrene (0.520 g, 1.761 mmol), 0.607 g of anhydrous K_2_CO_3_ (4.395 mmol) in dry MeCN (30 mL) was refluxed for 24 h under N_2_. After the evaporation of the solvent, the residue was partitioned between 0.1 M HCl and CH_2_Cl_2_. The organic layer was washed with water, dried over anhydrous Na_2_SO_4_, and concentrated. The oily residue was purified via column chromatography (SiO_2_, eluent hexane/AcOEt 8:1, v/v) to yield 3 (0.264 g, 46%) as a pale yellow solid. ^1^H-NMR (500 MHz, CDCl_3_) δ = 4.65 (d, *J* = 6.0 Hz, 2 H), 5.13 (d, *J* = 10.0 Hz, 1 H), 5.21 (dd, *J* = 17.5, 1.5 Hz, 1 H), 5.84 (s, 2 H), 5.95 (m, 1 H), 7.14 (t, *J* = 8.0 Hz, 1 H), 7.39 (dd, *J* = 8.0, 1.5 Hz, 1 H), 7.48 (dd, *J* = 8.0, 1.5 Hz, 1 H), 8.03-8.25 (m, 8 H), 8.36 (d, *J* = 9.0 Hz, 1 H), 10.46 (s, 1 H) ppm. ^13^C-NMR (CDCl3) δ = 70.09, 75.31, 118.9, 119.92, 120.46, 122.82, 124.14, 124.66, 124.94, 125.52, 125.60, 126.15, 126.88, 127.37, 127.84, 128.18, 129.07, 129.25, 130.30, 130.45, 130.69, 131.23, 133.77, 133.03, 152.06, 152.23, 190.42 ppm.

Synthesis of 3-(pyrenyl-1-methoxy) salicylaldehyde (4): In a 50 mL round-bottom flask, a stirred mixture of 2-allyloxy-3-(pyrenyl-1-methoxy) benzaldehyde 3 (0.237 g, 0.603 mmol), Pd(OAc)_2_ (4.49 mg, 0.020 mmol), 27.54 mg of PPh_3_ (0.105 mmol), Et_3_N (1.40 mL, 10.072 mmol, d = 0.728 g/mL), and 0.38 mL of HCO_2_H (d = 1.220 g/mL 10.072 mmol) in 80% EtOH (15 mL) was refluxed for 45 min. After the removal of the solvent, the residue was partitioned between CH_2_Cl_2_ and 0.1 M HCl. The organic layer was dried (Na_2_SO_4_) and concentrated. The product was purified via column chromatography (SiO_2_, eluent hexane/ AcOEt 8:1, v/v) to yield 4 (0.1593 g, 75%). ^1^H-NMR (500 MHz, CDCl_3_) δ = 5.91 (s, 2 H), 6.88 (t, *J* = 8.0 Hz, 1 H), 7.24 (m, 2 H), 8.02-8.24 (m, 8 H), 8.43 (d, *J* = 9.5 Hz, 1 H), 9.94 (s, 1 H), 11.11 (s, 1 H) ppm. ^13^C-NMR (CDCl3) δ = 70.44, 119.5, 121.27(x2), 122.2, 122.91, 124.64, 124.94, 125.45, 125.79, 126.04, 126.77, 127.37, 127.71, 128.16, 129.24, 129.39(x2), 130.76, 131.23, 133.59, 147.18, 152.73, 196.50 ppm.

Synthesis of Salen-Py (5): In a 50 mL round-bottom flask, a stirred mixture of 3-(pyrenyl-1-methoxy) salicylaldehyde **4** (0.117 g, 0.330 mmol), and (1*R*,2*R*)-1,2-diphenylethylenediamine (0.031 g, 0.148 mmol) in EtOH abs. (15 mL) was refluxed for 15 h under N_2_. After cooling, Salen–Py was collected via filtration (0.115 g, 88%), obtaining a yellow solid. ^1^H-NMR (500 MHz, Acetone-d_6_) δ = 5.12 (s, 2 H), 5.86 (d, *J* = 5.0 Hz, 4 H), 6.72 (m, 2 H), 6.98 (d, *J* = 7.5 Hz, 2 H), 7.20 (t, *J* = 7.0 Hz, 4 H), 7.27 (t, *J* = 7.5 Hz, 4 H), 7.40 (d, *J* = 7.5 Hz, 4 H), 8.05–8.29 (m, 16 H), 8.54 (d, *J* = 9.5 Hz, 2 H), 8.61 (s, 2 H), 13.70 (s, 2 H) ppm. ^13^C-NMR (CDCl3) δ = 70.33, 79.98, 80.09, 118.94, 119.66, 120.08, 124.26(x2), 125.11, 125.24(x2), 125.35, 125.96, 126.76, 127.79, 128.06(x2), 128.34, 128.76(x2), 128.94(x2), 129.92, 131.50, 131.60, 131.92, 131.99, 140.76, 147.77, 152.74, 167.31, 167.47 ppm.

Synthesis of the Zn–Salen–Py complex: In a 25 mL flask, (C_2_H_5_)_2_Zn (1M in hexane, Aldrich; 0.2 mL, 0.200 mmol) was added to a stirred solution of ligand 5 (30.6 mg, 3.473 × 10^−2^ mmol) in anhydrous toluene (10 mL). The mixture was stirred at room temperature for 24 h under N_2_ and was monitored via TLC (eluent: hexane/ AcOEt 7:3, v/v). The solvent was removed on a rotary evaporator under vacuum. The Zn–Salen–Py was obtained in nearly quantitative yields. ^1^H-NMR (500 MHz, DMSO-d_6_) δ = 5.15 (s, 2H), 5.46 (d, *J* = 7.5 Hz, 2H), 5.56 (d, *J* = 7.5 Hz, 2H), 7.09 (s, 2H), 7.16–7.23 (m, 2H), 7.26–7.34 (m, 4H), 7,40 (t, *J* = 7.5 Hz, 4H), 7.51 (d, *J* = 7.5 Hz, 3H), 7.66 (s, 2H), 7.77 (d, *J* = 7.5 Hz, 2H), 7.88–7.96 (m, 5H), 8.02 (t, *J* = 7.5 Hz, 2H) 8.09 (d, *J* = 7.5 Hz, 2H), 8.17 (d, *J* = 7.5 Hz, 2H), 8.18 (d, *J* = 7.5 Hz, 2H), 8.41 (t, *J* = 7.5 Hz, 4H) ppm.^13^C-NMR (CDCl3) δ = 69.83, 73.71, 112.28, 120.48, 120.58, 124.51, 125.07(x2), 125.89(x2), 126.88, 127.69, 127.81, 127.97, 128.15, 128.38, 128.59(x2), 129.27, 129.39(x2), 129.78, 131.03(x2), 131.45, 132.50, 142.11, 151.91(x2), 164.58, 170.71 ppm. HRMS: *m*/*z* 903.87 = [Salen-Py + Na]^+^ (calculated for C_62_H_44_N_2_O_4_Na^+^: 903.32) and *m*/*z* 965.77 [Zn–Salen–Py+Na]^+^ (calculated for Zn-C_62_H_42_N_2_O_4_Na^+^: 965.24).

## 4. Conclusions

In this work, we have presented the design and synthesis of a new chiral fluorescent Zn–Salen receptor bearing two pyrenyl arms located at the 3-3′ positions of the Salen backbone. Through fluorescence studies, we evaluated the recognition abilities of Zn–Salen–Py receptor for various environmentally relevant amines, including VOCs and biogenic amines. As expected for the design, the receptor showed a remarkable affinity for the tested amines. DFT calculations shed light on the recognition mechanism, thus supporting the high affinity values observed in solution. In addition, the presence of the chiral bridge on the receptor enabled the successful enantiodiscrimination of the two enantiomers of 1-(2-Naphtyl)ethylamine, showing outstanding enantioselectivity for the (*R*)-enantiomer. Finally, the calculated limits of detection, below the hazardous concentration of these analytes, pave the way for the use of this receptor in environmental monitoring and sensing applications.

## Figures and Tables

**Table 1:** Binding constant values (log*K*) and limit of detection (LOD) for the complexation of Zn–Salen–Py (1×10^−5^ M) with selected amines in methanol.

**Entry**	**Guest**	**Binding Constant (log*K*) ^a^**	**LOD (µM) ^b^**
**1 ^c^**	Ethylamine	5.34 ± 0.01	0.22
**2 ^c^**	Propylamine	6.03 ± 0.01	0.21
**3 ^c^**	Butylamine	6.21 ± 0.01	0.23
**4 ^c^**	Hexylamine	6.45 ± 0.02	0.15
**5 ^c^**	Phenylethylamine	6.39 ± 0.02	0.35
**6 ^c^**	Phenylpropylamine	6.45 ± 0.01	0.27
**7 ^c^**	Phenylbutylamine	6.49 ± 0.01	0.22
**8 ^c^**	Tyramine	5.90 ± 0.01	0.13
**9 ^c^**	Methoxytyramine	5.69 ± 0.01	0.17
**10 ^d^**	*R*(+)-1-(2-Naphtyl)ethylamine	7.59 ± 0.07	0.12
**11 ^d^**	*S*(−)-1-(2-Naphtyl)ethylamine.	5.61 ± 0.02	0.28

^a^ Calculated using HypSpec software; ^b^ calculated using 3σ/k formula, where k is the slope of the I/I0 vs. the guest concentration curve, and σ is the standard deviation of the blank; ^c^ calculated at λ_ex_ = 350 nm; ^d^ calculated at λ_ex_ = 330 nm.

**Table 2:** Complexation energy (E_complex_) of the host–guest complex between Zn–Salen–Py and the selected amines, calculated in the gas phase at the B3LYP/6-31G(d,p) level of theory.

**Entry**	**Guest**	**Complexation Energy (E_complex_)**
**1**	Ethylamine	19.2
**2**	Hexylamine	17.9 ^a^
**3**	Phenylethylamine	16.2
**4**	Phenylbutylamine	15.8 ^a^
**5**	*R*(+)-1-(2-Naphtyl)ethylamine	14.9
**6**	*S*(−)-1-(2-Naphtyl)ethylamine.	10.9 ^a^

^a^ Averaged value calculated taking into account the Boltzmann distribution of the main conformers (see the Supplementary Materials).

## Data Availability

All the synthesized compounds, as well as the data used in this work, can be obtained from the authors upon formal request.

## Supplementary Materials

The following supporting information can be downloaded at: https://www.mdpi.com/article/10.3390/molecules29235796/s1, Figure S1: 1H NMR spectrum of compound 2 in CDCl3; Figure S2: 1H-NMR of the compound 3 in CDCl3; Figure S3: 13C-NMR of the compound 3 in CDCl3; Figure S4: 1H-NMR of the compound 4 in CDCl3; Figure S5: 13C-NMR of the compound 4 in CDCl3; Figure S6: 1H-NMR of the compound Salen-Py in Acetone-d6; Figure S7: 13C-NMR of the compound Salen-Py in Acetone-d6; Figure S8: 1H-NMR of the compound Zn-Salen-Py in DMSO-d6; Figure S9: 13C-NMR of the compound Zn-Salen-Py in DMSO-d6; Figure S10: ESI(+)-MS spectrum of Zn-Salen-Py; Figure S11: Isotopic distribution of Zn-Salen-Py; Figure S12: Uv-Vis spectra of Zn-Salen-Py in methanol; Figure S13: Calibration curves of Zn-Salen-Py for the epsilon calculation; Figure S14: Fluorescence spectra of Zn-Salen-Py; Figure S15: Fluorescence titration of Zn-Salen-Py vs. Ethylamine; Figure S16: Fluorescence titration of Zn-Salen-Py vs. Propylamine; Figure S17: Fluorescence titration of Zn-Salen-Py vs. Butylamine; Figure S18: Fluorescence titration of Zn-Salen-Py vs. Hexylamine; Figure S19: Fluorescence titration of Zn-Salen-Py vs. Phenylethylamine; Figure S20: Fluorescence titration of Zn-Salen-Py vs. Phenylpropylamine; Figure S21: Fluorescence titration of Zn-Salen-Py vs. Phenylbutylamine; Figure S22: Fluorescence titration of Zn-Salen-Py vs. Tyramine; Figure S23: Fluorescence titration of Zn-Salen-Py vs. Methoxytyramine; Figure S24: Fluorescence titration of Zn-Salen-Py vs R-(+)-1-(2Naphtyl)ethylamine; Figure S25: Fluorescence titration of Zn-Salen-Py vs. R(+)-1-(2-Naphtyl)ethylamine@Zn-Salen-Py; Figure S26. Conformational study on Zn-Salen-Py; Figure S27. Optimized geometry for the phenylethylamine@Zn-Salen-Py; Figure S28. Optimized geometry for the phenylbutylamine@Zn-Salen-Py; Figure S29. Optimized geometry for the ethylamine@Zn-Salen-Py; Figure S30. Optimized geometry for the hexylamine@Zn-Salen-Py; Figure S31. Optimized geometry for the R(+)-1-(2-Naphtyl)ethylamine@Zn-Salen-Py; Figure S32: Optimized geometry for the S(-)-1-(2-Naphtyl)ethylamine@Zn-Salen-Py.
