# Supplementary material for: Detection of VOCs and Biogenic Amines Through Luminescent Zn–Salen Complex-Tethered Pyrenyl Arms
Source: Molecules. 2024 Dec 8;29(23):5796. doi: 10.3390/molecules29235796 (PMC11643397; doi:10.3390/molecules29235796)
Supplement: Supplementary file 1 [file molecules-29-05796-s001.zip › molecules-3332602-supplementary.pdf]

Detection of VOC and Biogenic Amines by a Luminescent Zn-Salen Complex Tethered Pyrenyl arms

Roberta Puglisi,[a] Caterina Testa,[a] Sara Scuderi,[a] Valentina Greco,[a] Giuseppe Trusso Sfrazzetto,[a] Manuel Petroselli,[b] and Andrea Pappalardo[a]

[a] Dipartimento di Scienze Chimiche, Università degli Studi di Catania, Viale A. Doria 6, 95125 Catania, Italy.

[b] Center for Supramolecular Chemistry and Catalysis and Department of Chemistry, College of Science, Shanghai University, Shanghai 200444, China. Current Position: Institute of Chemical Research of Catalonia (ICIQ), Av. Països Catalans 16, 43007 Tarragona, Spain.

SUPPLEMENTARY MATERIALS

Table of Contents

NMR SPECTRA 3

Figure S1. ^{1}H NMR spectrum of compound **2** in $CDCl_3$**.** 3

Figure S2.^{1}H-NMR of the compound **3** in $CDCl_3$. 3

Figure S3.^{13}C-NMR of the compound **3** in $CDCl_3$. 4

Figure S4. ^{1}H-NMR of the compound **4** in $CDCl_3$. 4

Figure S5.^{13}C-NMR of the compound **4** in $CDCl_3$. 5

Figure S6. ^{1}H-NMR of the compound **Salen-Py** in Acetone-d_6. 5

Figure S7.^{13}C-NMR of the compound **Salen-Py** in Acetone-d_6. 6

Figure S8. ^{1}H-NMR of the compound **Zn-Salen-Py** in DMSO-d_6. 6

Figure S9.^{13}C-NMR of the compound **Zn-Salen-Py** in DMSO-d_6. 7

MASS SPECTROMETRY ANALISYS 7

Figure S10. (a) ESI(+)-MS spectrum of **Zn-Salen-Py**. 7

Figure S11. Isotopic distribution of **Zn-Salen-Py** 8

OPTICAL CHARACTERIZATION 8

Figure S12. Uv-Vis spectra of **Zn-Salen-Py** in methanol. 8

Figure S13. Calibration curves of **Zn-Salen-Py** for the epsilon calculation. 9

Figure S14. Fluorescence spectra of **Zn-Salen-Py** 9

Figure S15. Fluorescence titration of **Zn-Salen-Py** *vs* Ethylamine.. 10

Figure S16. Fluorescence titration of **Zn-Salen-Py** *vs* Propylamine.. 10

Figure S17. Fluorescence titration of **Zn-Salen-Py** *vs* Butylamine ....**Error! Bookmark not defined.**

Figure S18. Fluorescence titration of **Zn-Salen-Py** *vs* Hexylamine...........**Error! Bookmark not defined.**

Figure S19. Fluorescence titration of **Zn-Salen-Py** *vs* Phenylethylamine .**Error! Bookmark not defined.**

Figure S20. Fluorescence titration of **Zn-Salen-Py** *vs* Phenylpropylamine............ **Error! Bookmark not defined.**

Figure S21. Fluorescence titration of **Zn-Salen-Py** *vs* Phenylbutylamine.**Error! Bookmark not defined.**

Figure S22. Fluorescence titration of **Zn-Salen-Py** *vs* Tyramine ....**Error! Bookmark not defined.**

Figure S23. Fluorescence titration of **Zn-Salen-Py** *vs* Methoxytyramine .**Error! Bookmark not defined.**

Figure S24. Fluorescence titration of **Zn-Salen-Py** *vs* R-(+)-1-(2Naphtyl)ethylamine ..**Error! Bookmark not defined.**

Figure S25. Fluorescence titration of **Zn-Salen-Py** *vs* S-(+)-1-(2Naphtyl)ethylamine...**Error! Bookmark not defined.**

COMPUTATIONAL DATA 15

Figure S26. Conformational study on **Zn-Salen-Py** ....**Error! Bookmark not defined.**

Figure S27. Optimized geometry for the **phenylethylamine@Zn-Salen-Py** .... **Error! Bookmark not defined.**

Figure S28. Optimized geometry for the **phenylbutylamine@Zn-Salen-Py** .... **Error! Bookmark not defined.**

Figure S29. Optimized geometry for the **ethylamine@Zn-Salen-Py** ......**Error! Bookmark not defined.**

Figure S30. Optimized geometry for the **hexylamine@Zn-Salen-Py** .....**Error! Bookmark not defined.**

Figure S31. Optimized geometry for the ***R*(+)-1-(2-Naphtyl)ethylamine@Zn-Salen-Py** ....**Error! Bookmark not defined.**

Figure S32. Optimized geometry for the ***S*(-)-1-(2-Naphtyl)ethylamine@Zn-Salen-Py** ....**Error! Bookmark not defined.**

NMR SPECTRA

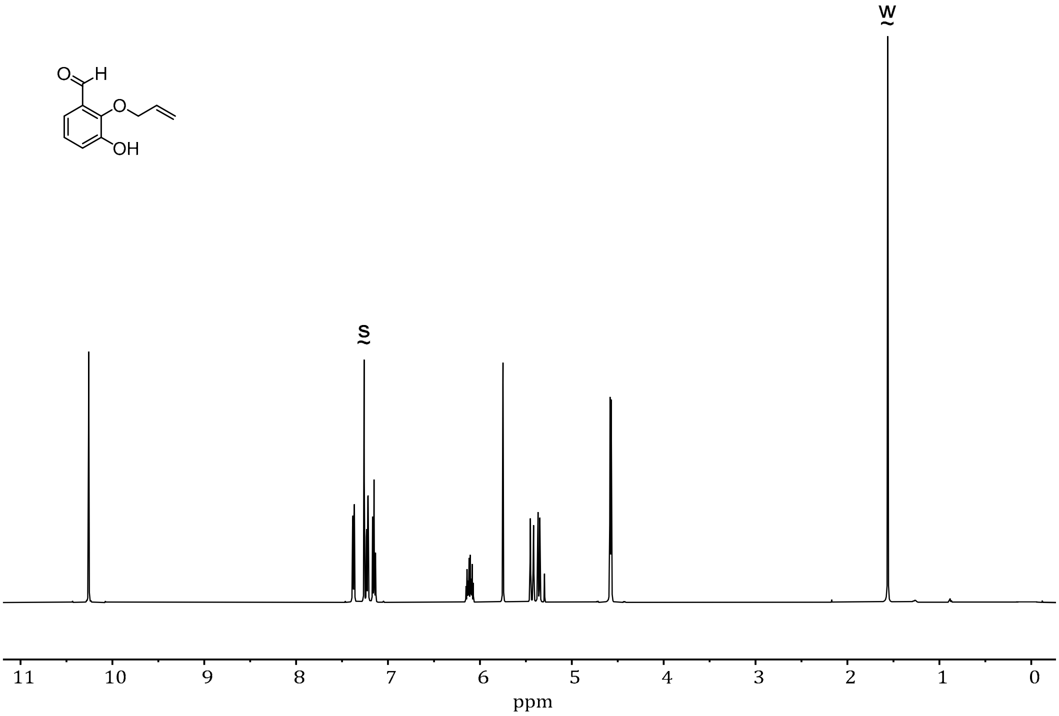

Figure S1. ^{1}H NMR spectrum of compound **2** in $CDCl_3$**.**

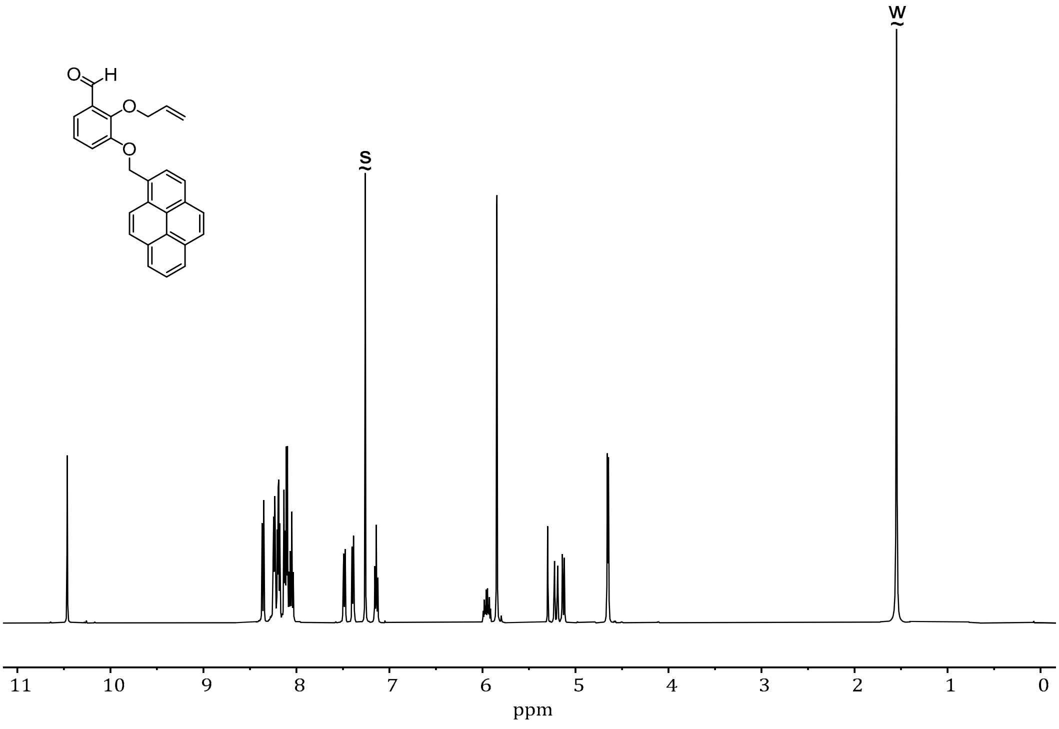

Figure S2.^{1}H-NMR of the compound **3** in $CDCl_3$.

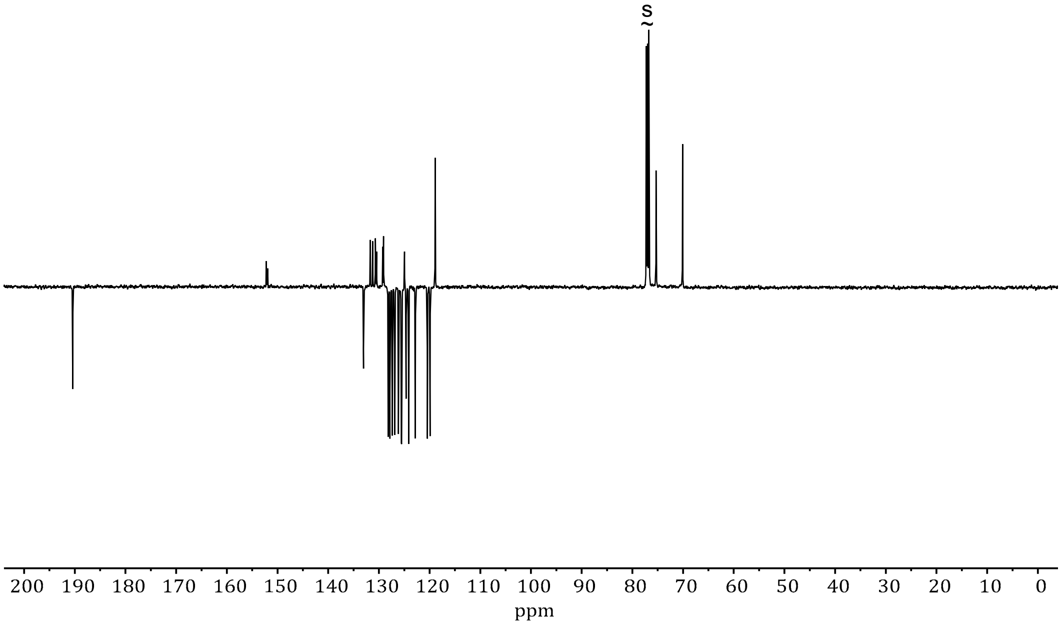

Figure S3. ^{13}C-NMR of the compound **3** in $CDCl_3$.

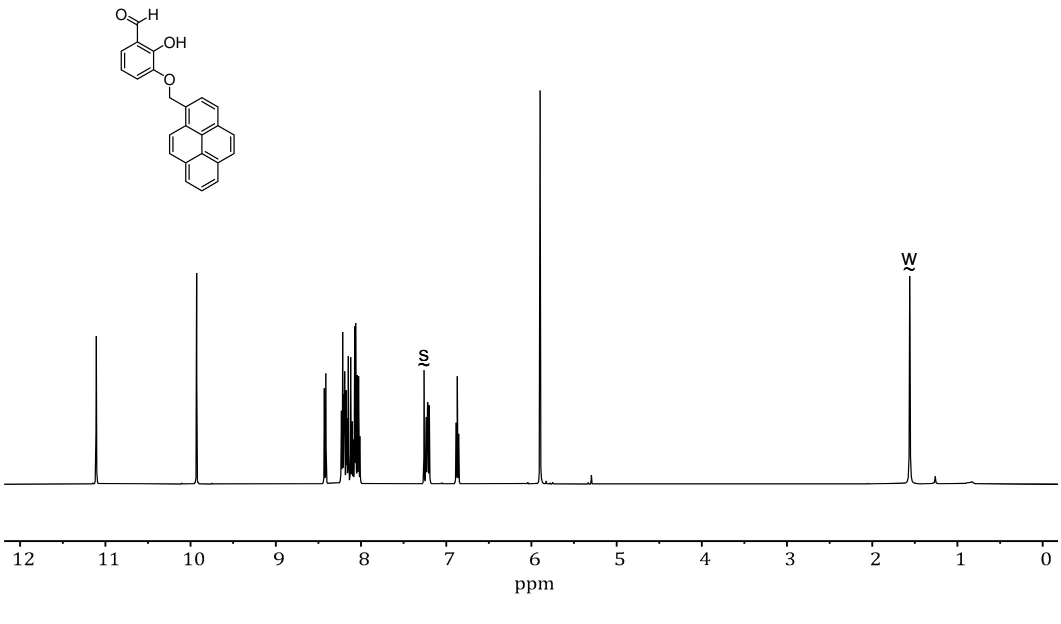

Figure S4. ^{1}H-NMR of the compound **4** in $CDCl_3$.

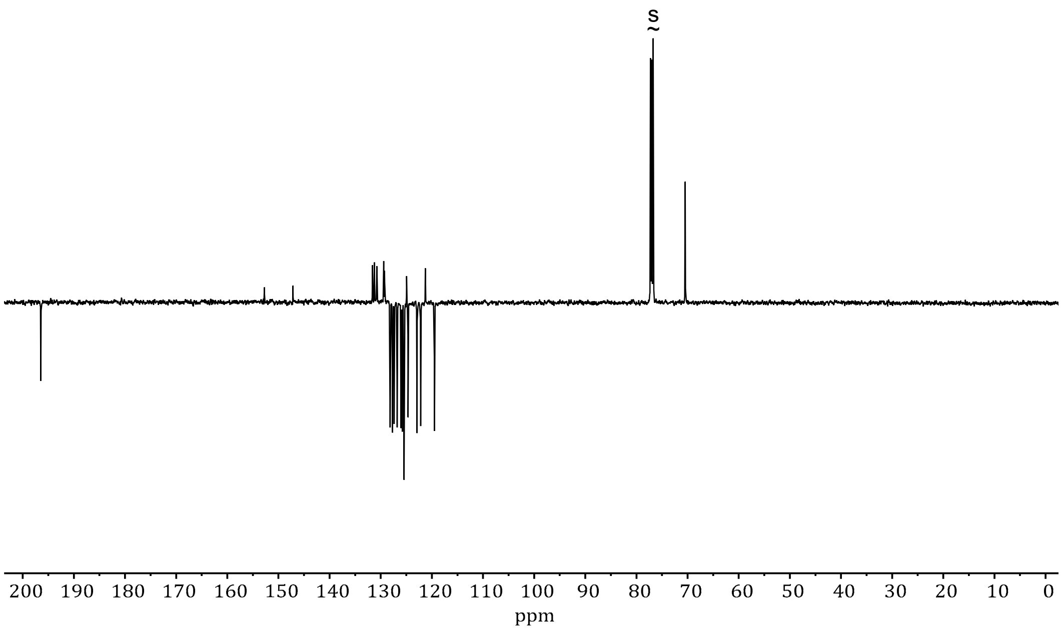

Figure S5.^{13}C-NMR of the compound **4** in $CDCl_3$.

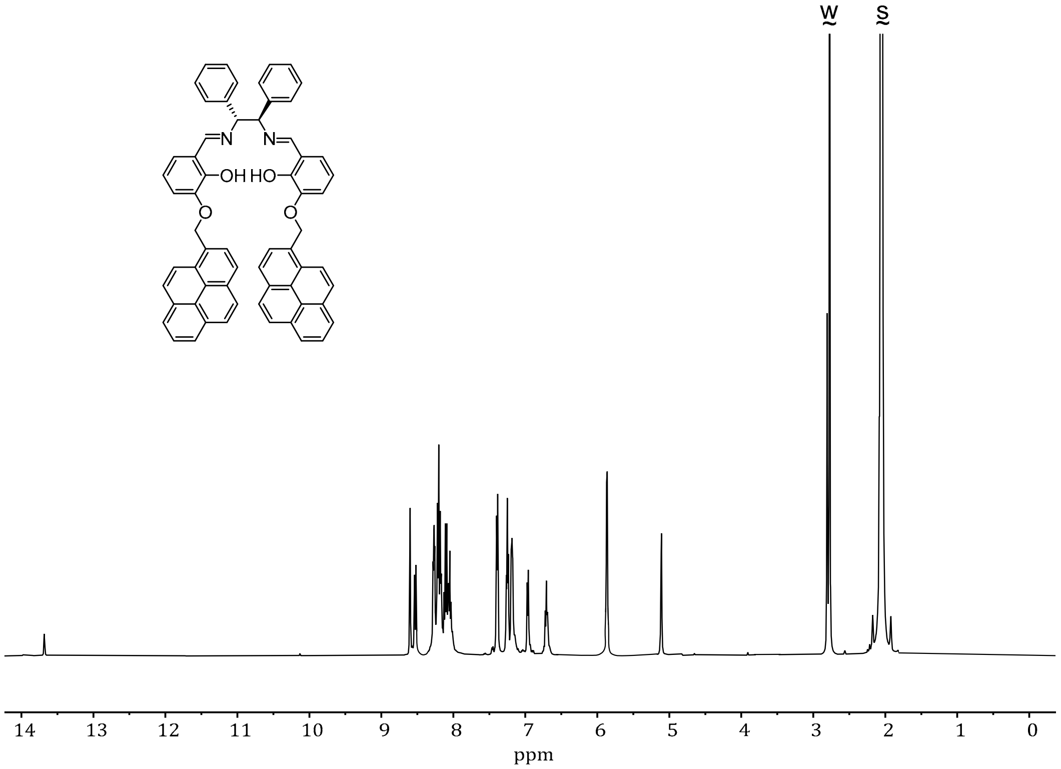

Figure S6. ^{1}H-NMR of the compound **Salen-Py** in Acetone-d_6.

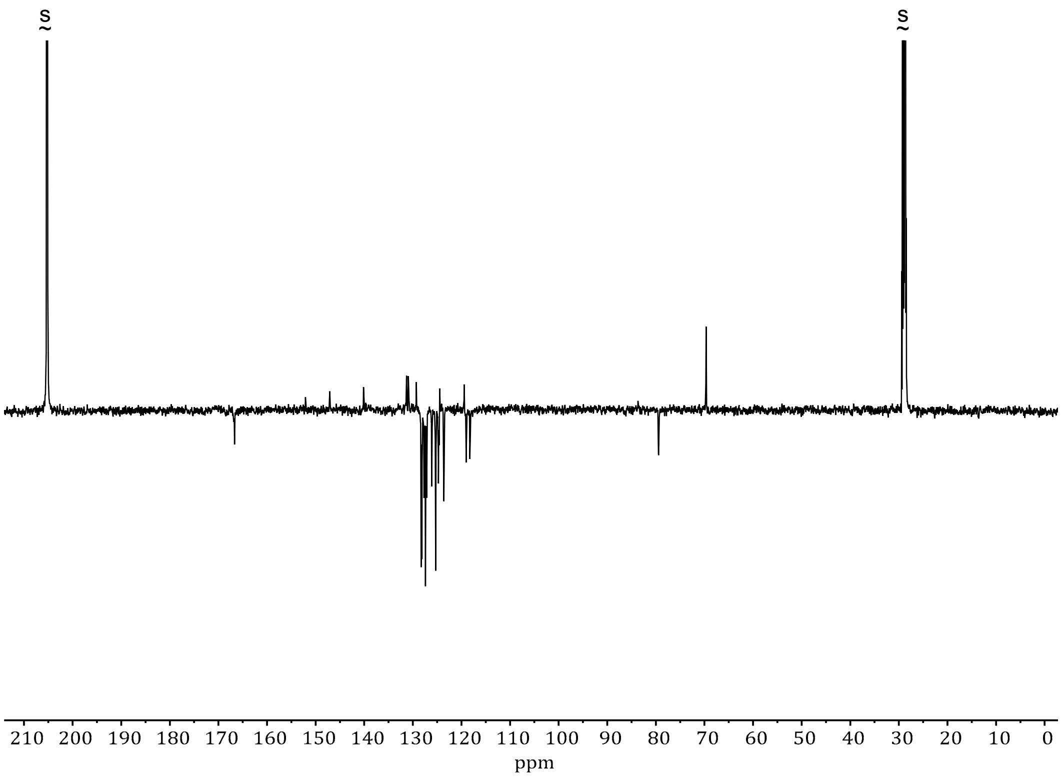

Figure S7.^{13}C-NMR of the compound **Salen-Py** in Acetone-d$_6$.

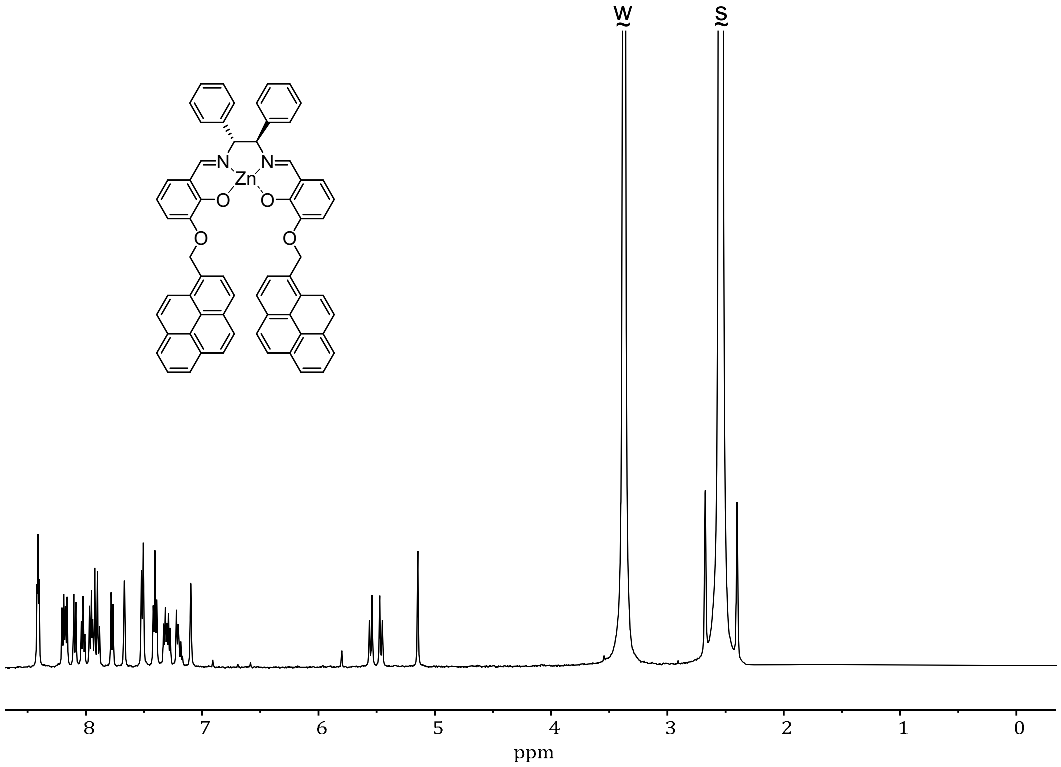

Figure S8. ^{1}H-NMR of the compound **Zn-Salen-Py** in DMSO-d$_6$.

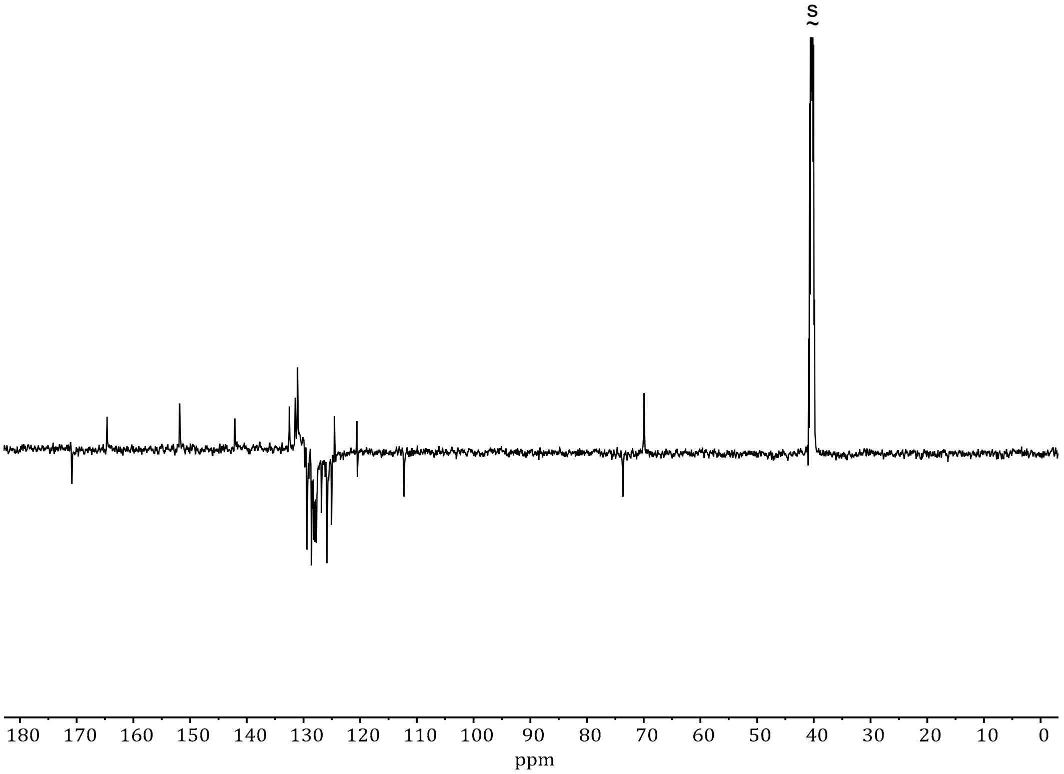

Figure S9. ^{13}C-NMR of the compound **Zn-Salen-Py** in DMSO-d_6.

MASS SPECTROMETRY ANALISYS

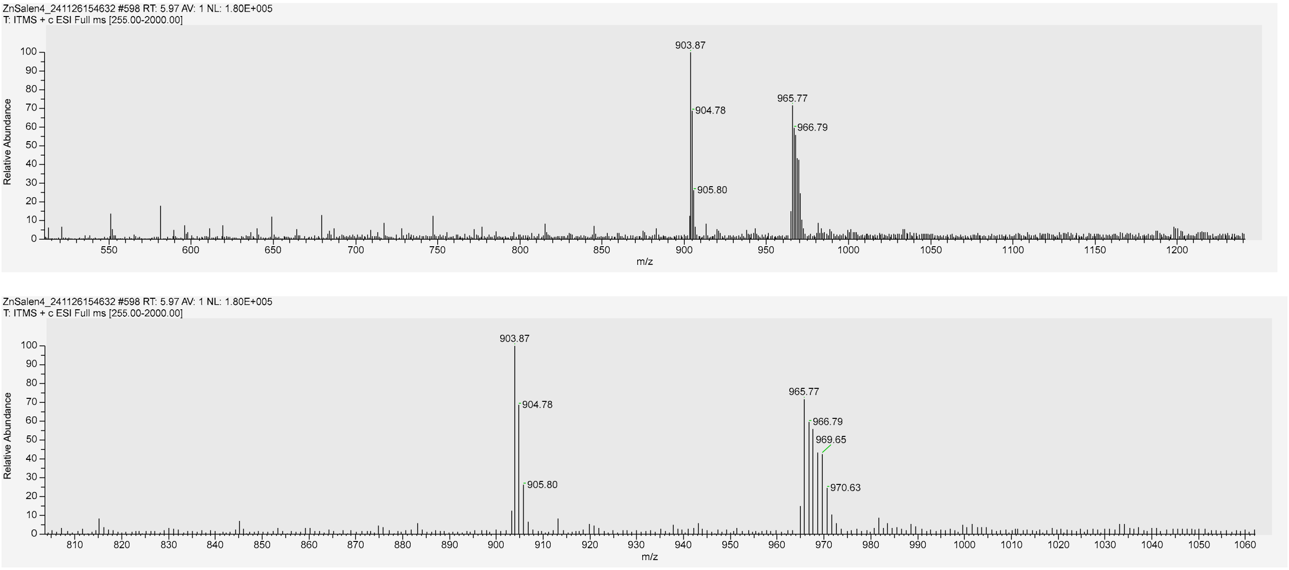

Figure S10. (a) ESI(+)-MS spectrum of **Zn-Salen-Py** ($1x10^{-6}$M in methanol), *m/z* 903.87 = [**Salen-Py + Na**]$^+$ (calculated for $C_{62}H_{44}N_2O_4Na^+$: 903.32) and *m/z* 965.77 [**Zn-Salen-Py**+Na]$^+$ (calculated for Zn-$C_{62}H_{42}N_2O_4Na^+$: 965.24)**; (b)** magnification for 800-1060 *m/z* region.

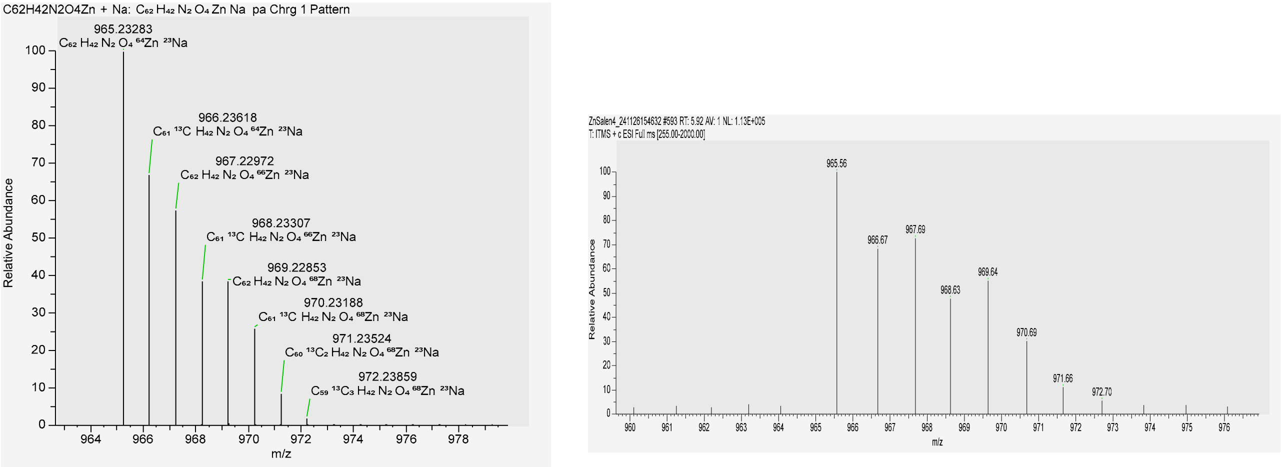

Figure S11. Isotopic distribution of **Zn-Salen-Py** in sodiate form (Zn-$C_{62}H_{42}N_2O_4Na^+$; [**Zn-Salen-Py**+Na]$^+$: left) simulation by Xcalibur software, right) experimental.

OPTICAL CHARACTERIZATION

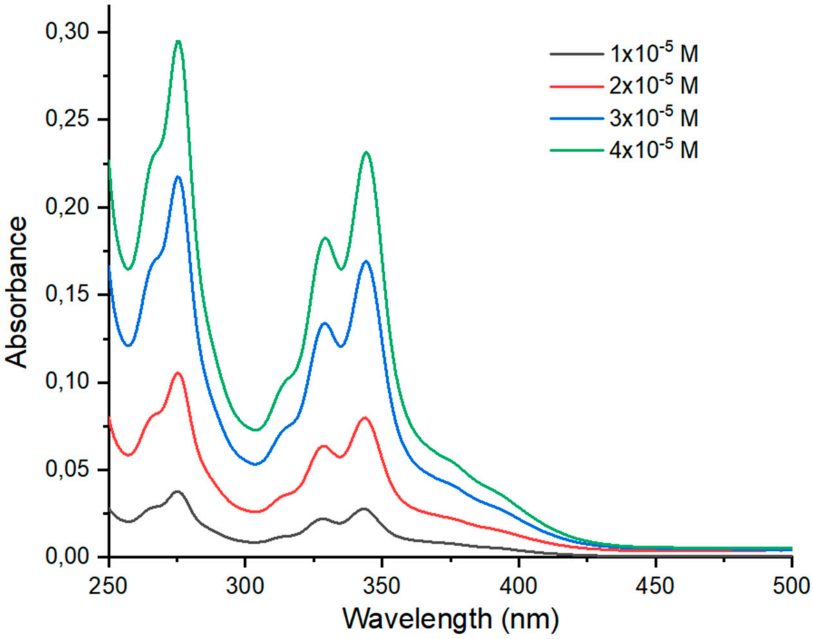

Figure S12. Uv-Vis spectra of **Zn-Salen-Py** in methanol at different concentrations.

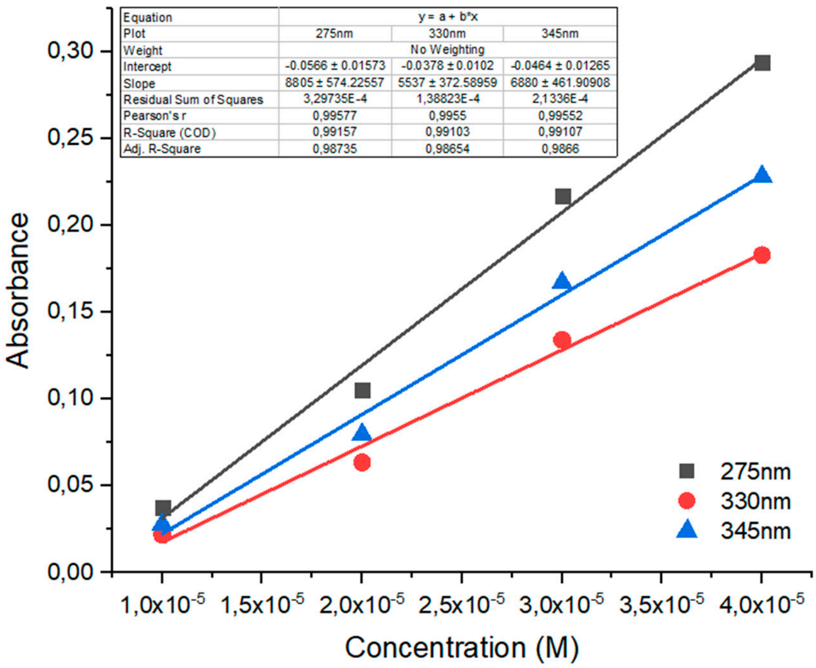

Figure S13. Calibration curves of **Zn-Salen-Py** obtained from the absorption spectra at different concentrations (275 nm ε = 8805 $cm^{-1}M^{-1}$, 330 nm ε = 5537 $cm^{-1}M^{-1}$, 350 nm ε = 6880 $cm^{-1}M^{-1}$) for the epsilon calculation.

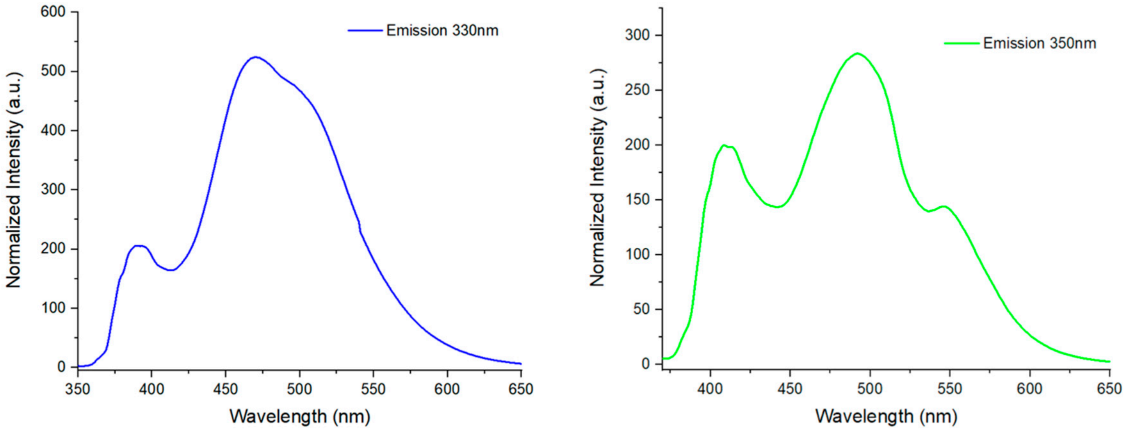

Figure S14. Fluorescence spectra of **Zn-Salen-Py** at different excitation wavelengths. (λ_{ex} = 330nm and λ_{ex} = 350nm)

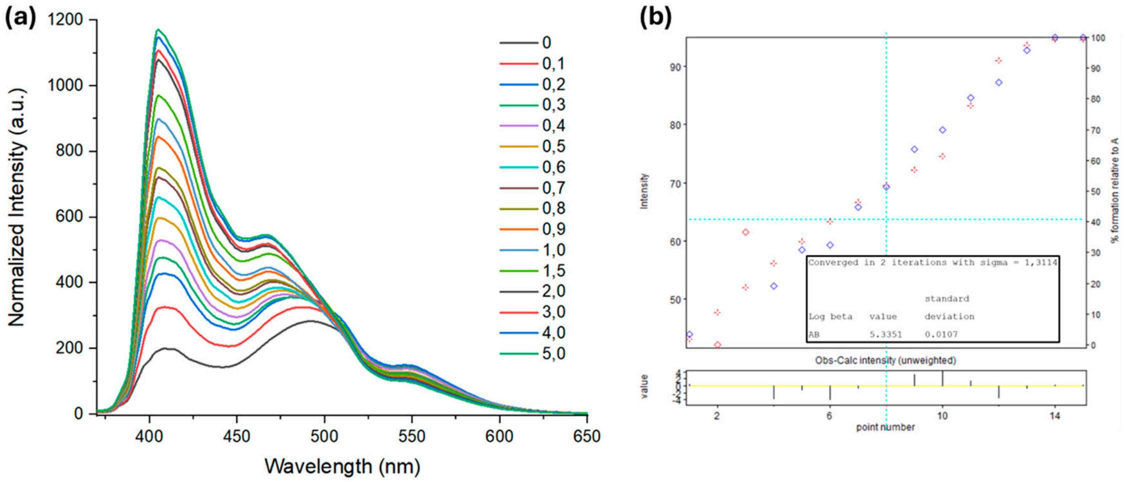

Figure S15. (a) Fluorescence titration of **Zn-Salen-Py** ($1x10^{-5}$M in methanol) at λ_{ex} = 350nm with Ethylamine (from 0 to 5 equivalents); **(b)** HypSpec plot of fluorescence titration (blue points are experimental values, red points represent theoretical curve). **Inset**: HypSpec output files with Log value and standard deviation.

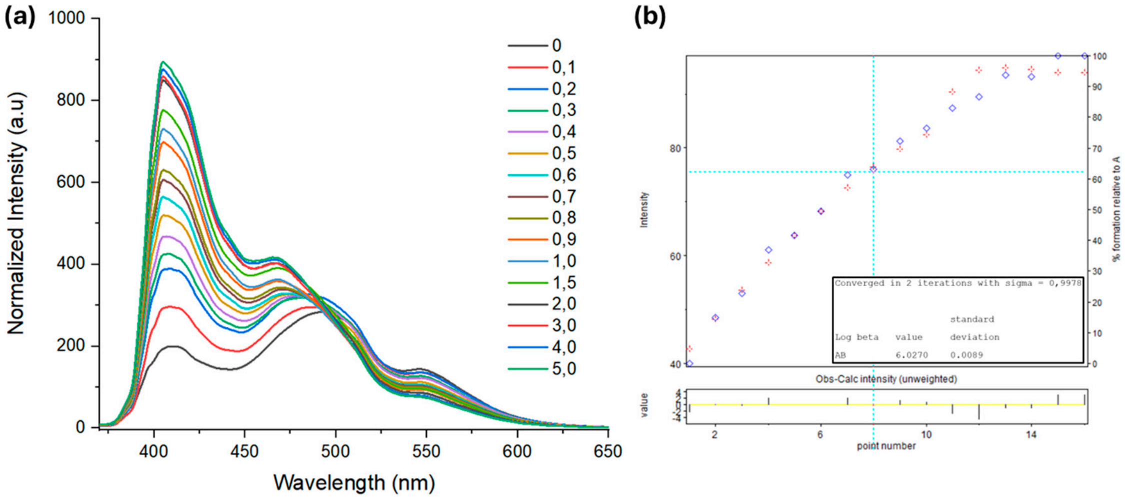

Figure S16. (a) Fluorescence titration of **Zn-Salen-Py** ($1x10^{-5}$M in methanol) at λ_{ex} = 350nm with Propylamine (from 0 to 5 equivalents); **(b)** HypSpec plot of fluorescence titration (blue points are experimental values, red points represent theoretical curve). **Inset**: HypSpec output files with Log value and standard deviation.

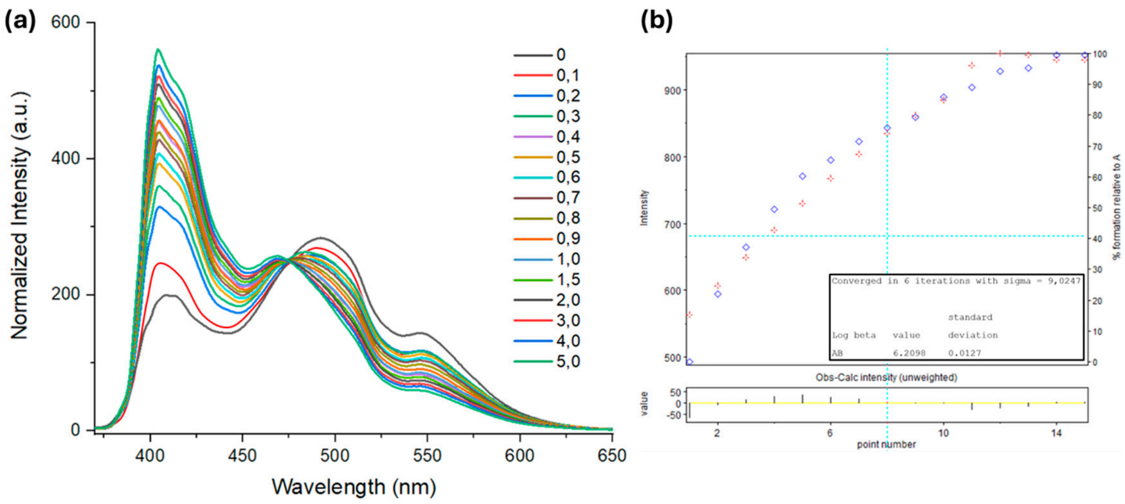

Figure S17. (a) Fluorescence titration of **Zn-Salen-Py** ($1x10^{-5}$M in methanol) at λ_{ex} = 350nm with Butylamine (from 0 to 5 equivalents); **(b)** HypSpec plot of fluorescence titration (blue points are experimental values, red points represent theoretical curve). **Inset**: HypSpec output files with Log value and standard deviation.

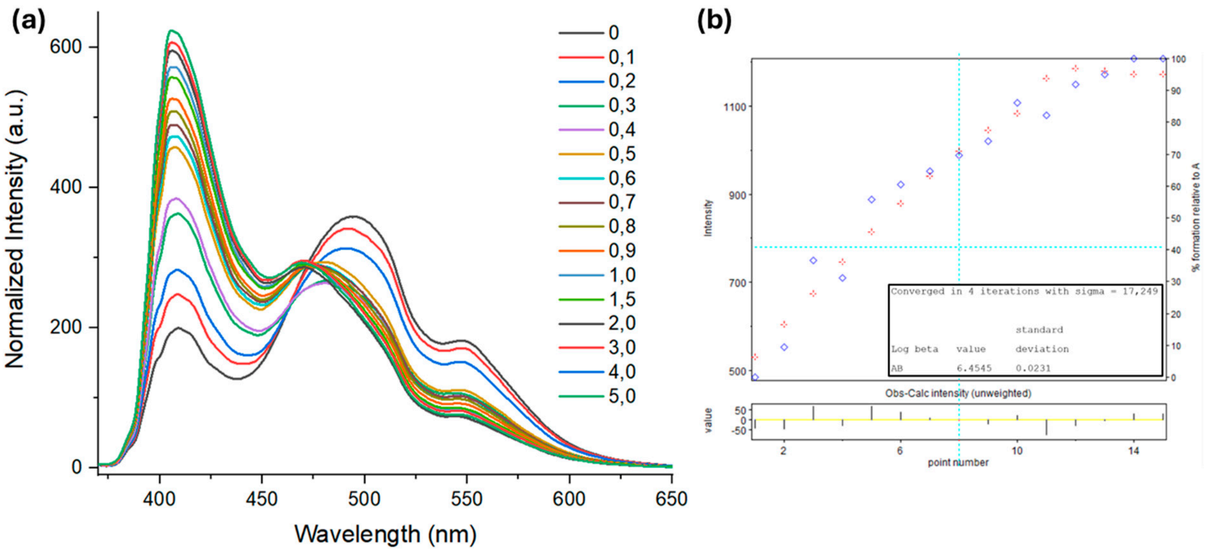

Figure S18. (a) Fluorescence titration of **Zn-Salen-Py** ($1x10^{-5}$M in methanol) at λ_{ex} = 350nm with Hexylamine (from 0 to 5 equivalents); **(b)** HypSpec plot of fluorescence titration (blue points are experimental values, red points represent theoretical curve). **Inset**: HypSpec output files with Log value and standard deviation.

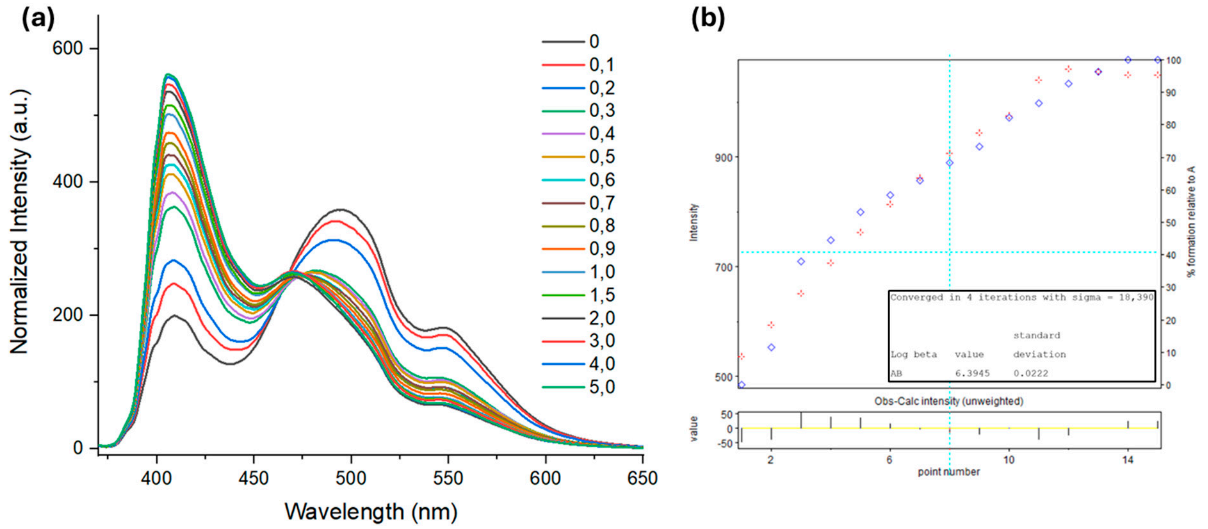

Figure S19. (a) Fluorescence titration of **Zn-Salen-Py** ($1x10^{-5}$M in methanol) at λ_{ex} = 350nm with Phenylethylamine (from 0 to 5 equivalents); **(b)** HypSpec plot of fluorescence titration (blue points are experimental values, red points represent theoretical curve). **Inset**: HypSpec output files with Log value and standard deviation.

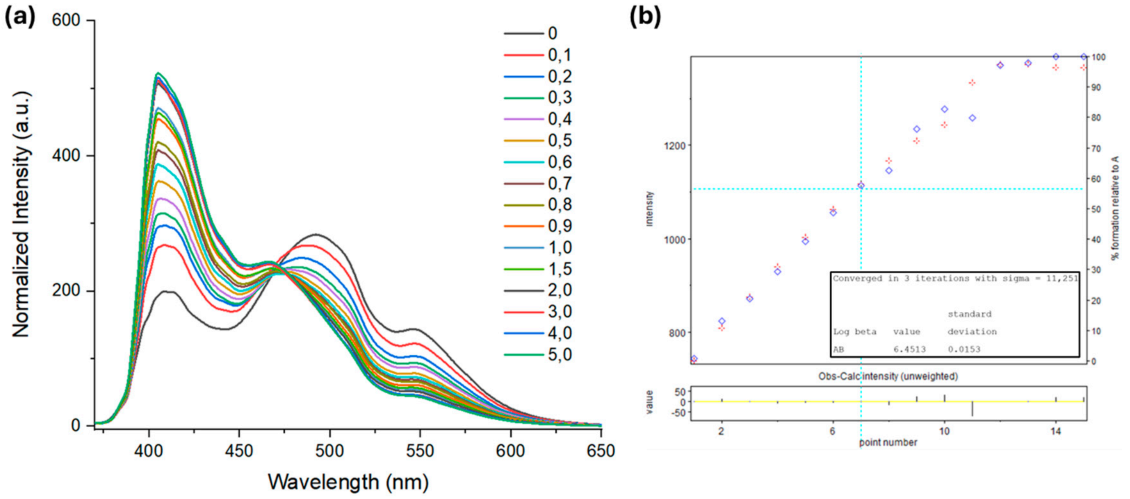

Figure S20. (a) Fluorescence titration of **Zn-Salen-Py** ($1x10^{-5}$M in methanol) at λ_{ex} = 350nm with Phenylpropylamine (from 0 to 5 equivalents); **(b)** HypSpec plot of fluorescence titration (blue points are experimental values, red points represent theoretical curve). **Inset**: HypSpec output files with Log value and standard deviation.

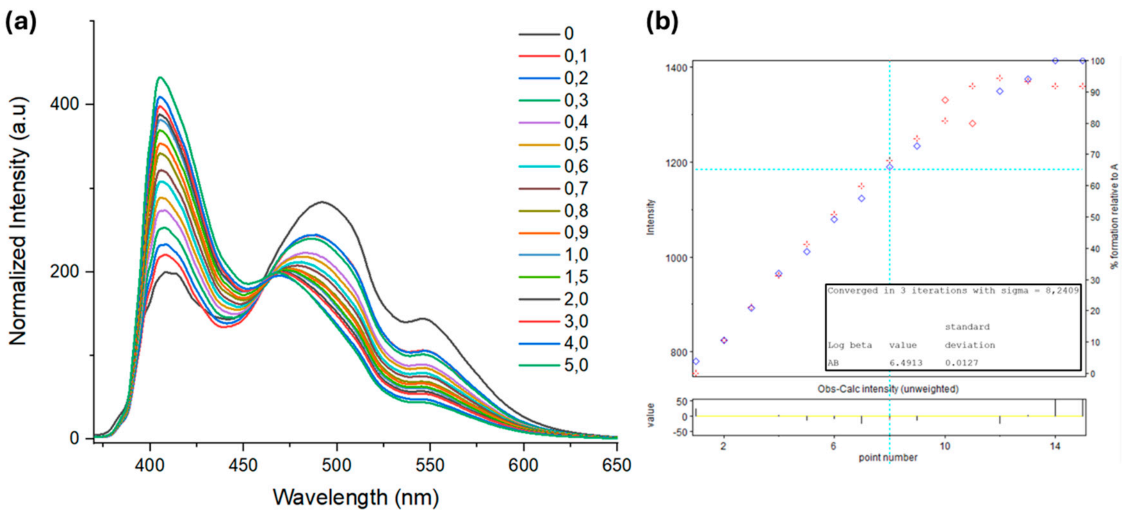

Figure S21. (a) Fluorescence titration of **Zn-Salen-Py** (1x10^{-5}M in methanol) at λ_{ex} = 350nm with Phenylbutylamine (from 0 to 5 equivalents); **(b)** HypSpec plot of fluorescence titration (blue points are experimental values, red points represent theoretical curve). **Inset**: HypSpec output files with Log value and standard deviation.

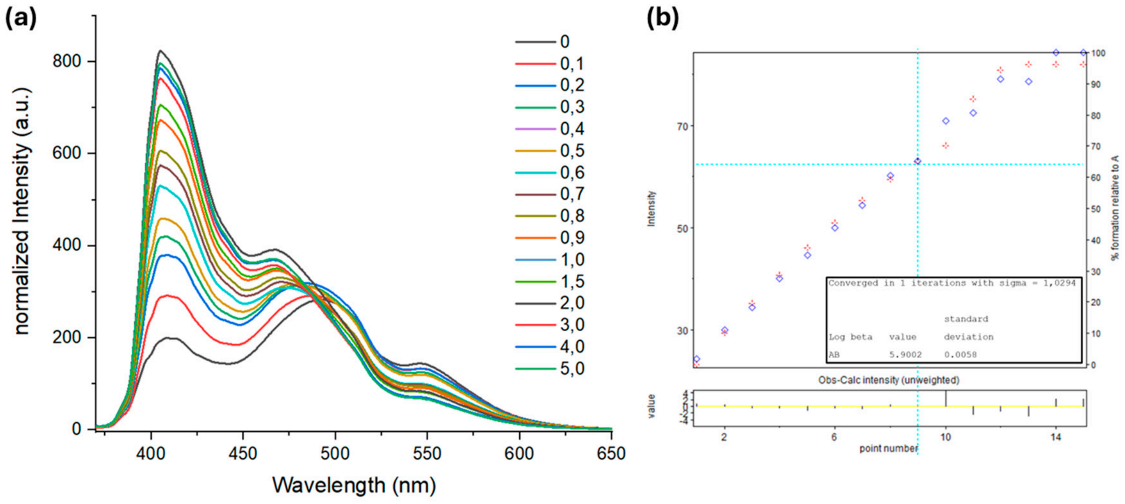

Figure S22. (a) Fluorescence titration of **Zn-Salen-Py** (1x10^{-5}M in methanol) at λ_{ex} = 350nm with Tyramine (from 0 to 5 equivalents); **(b)** HypSpec plot of fluorescence titration (blue points are experimental values, red points represent theoretical curve). **Inset**: HypSpec output files with Log value and standard deviation.

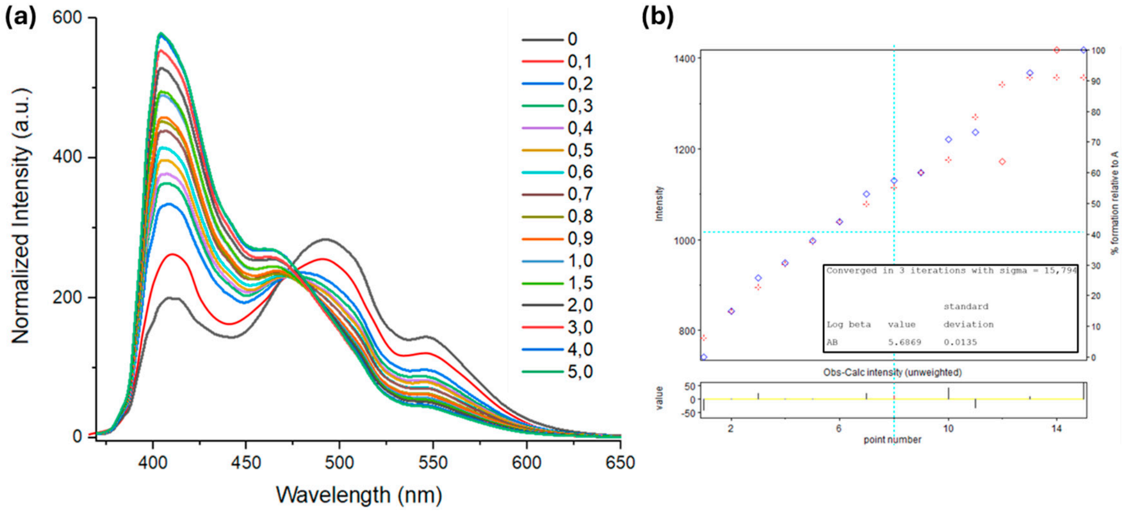

Figure S23. (a) Fluorescence titration of **Zn-Salen-Py** (1x10^{-5}M in methanol) at λ_{ex} = 350nm with Methoxytyramine (from 0 to 5 equivalents); **(b)** HypSpec plot of fluorescence titration (blue points are experimental values, red points represent theoretical curve). **Inset**: HypSpec output files with Log value and standard deviation.

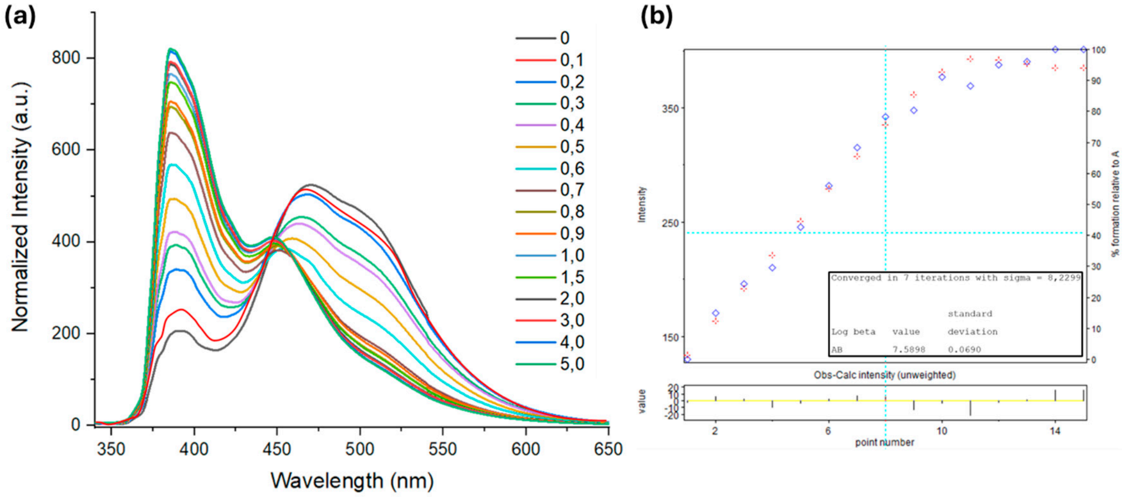

Figure S24. (a) Fluorescence titration of **Zn-Salen-Py** (1x10^{-5}M in methanol) at λ_{ex} = 330nm with *R*-(+)-1-(2-Naphtyl)ethylamine (from 0 to 5 equivalents); **(b)** HypSpec plot of fluorescence titration (blue points are experimental values, red points represent theoretical curve). **Inset**: HypSpec output files with Log value and standard deviation.

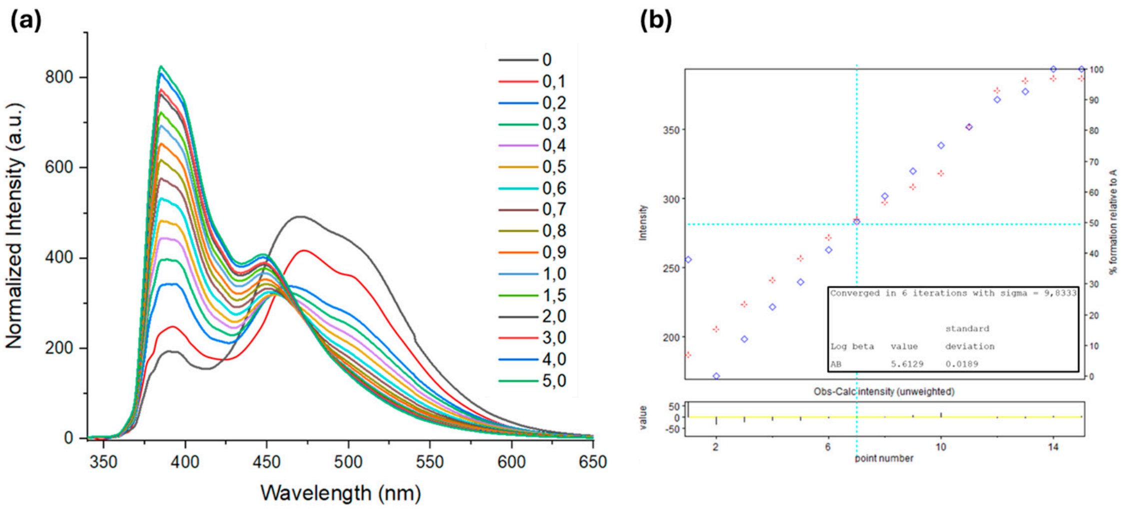

Figure S25. (a) Fluorescence titration of **Zn-Salen-Py** ($1x10^{-5}$M in methanol) at λ_{ex} = 330nm with *S*-(-)-1-(2-Naphtyl)ethylamine (from 0 to 5 equivalents); **(b)** HypSpec plot of fluorescence titration (blue points are experimental values, red points represent theoretical curve). Inset: HypSpec output files with Log value and standard deviation.

COMPUTATIONAL DATA

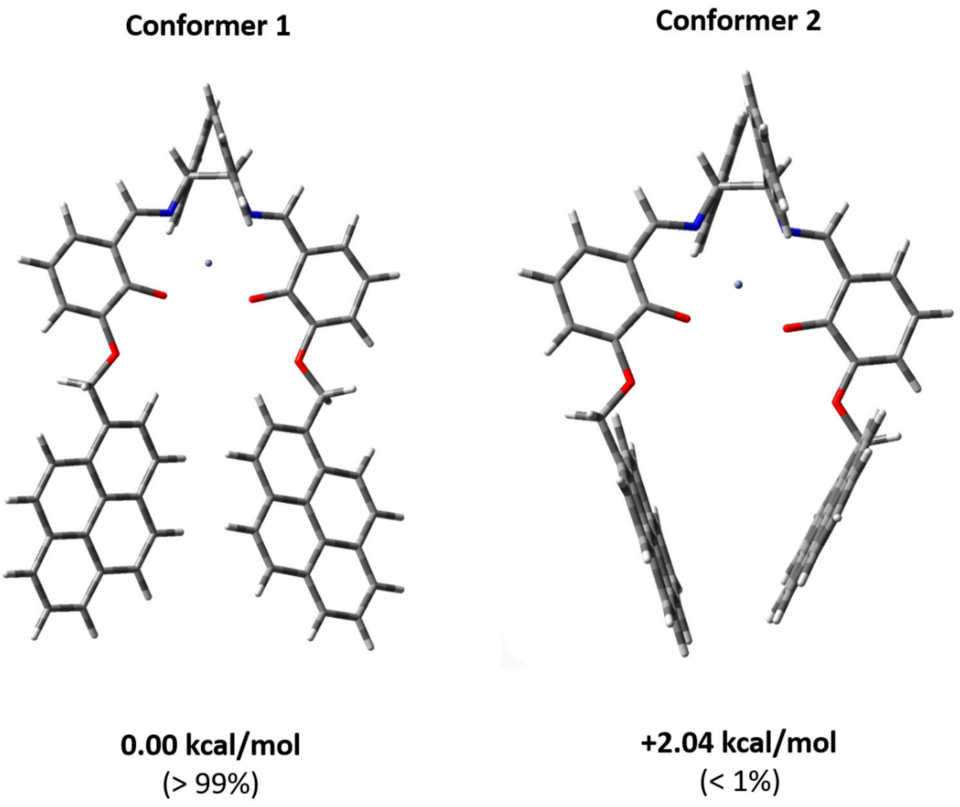

Figure S26. Conformational study on **Zn-Salen-Py** host in gas phase at B3LYP/6-31G(d,p).

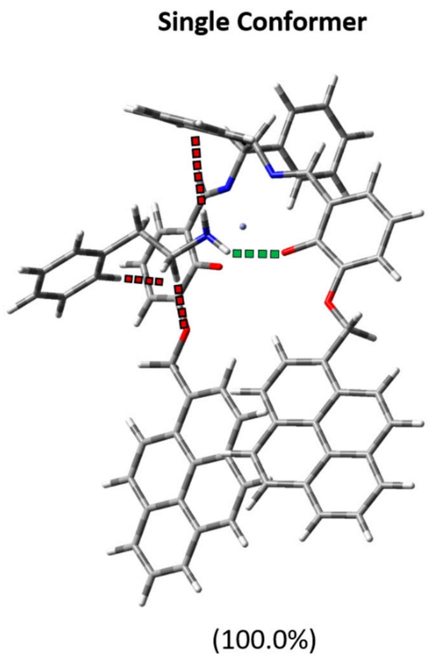

Figure S27. Optimized geometry for the **phenylethylamine@Zn-Salen-Py** complex in gas phase at B3LYP/6-31G(d,p). NH-O interactions are marked in green while NH-π, CH-π and CH-O interactions in red. Boltzmann distribution is reported between brakets.

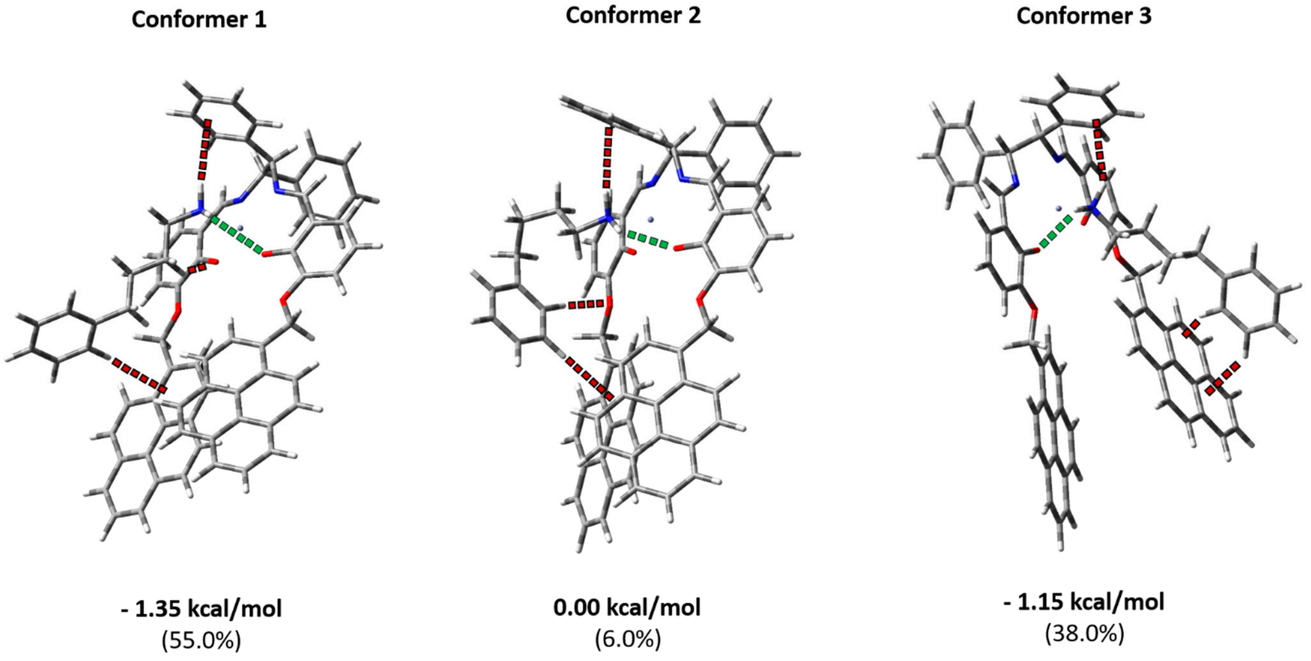

Figure S28. Optimized geometry for the **phenylbutylamine@Zn-Salen-Py** complex in gas phase at B3LYP/6-31G(d,p). NH-O interactions are marked in green while NH-π, CH-π and CH-O interactions in red. Boltzmann distribution is reported between brakets.

Single Conformer

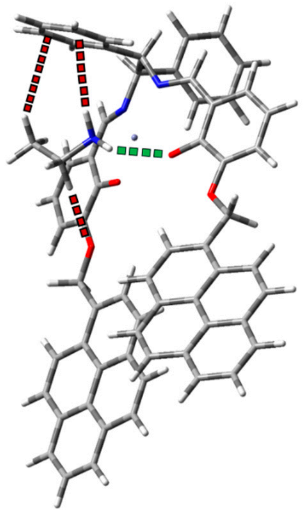

(100.0%)

Figure S29. Optimized geometry for the **ethylamine@Zn-Salen-Py** complex in gas phase at B3LYP/6-31G(d,p). NH-O interactions are marked in green while NH-π, CH-π and CH-O interactions in red. Boltzmann distribution is reported between brakets.

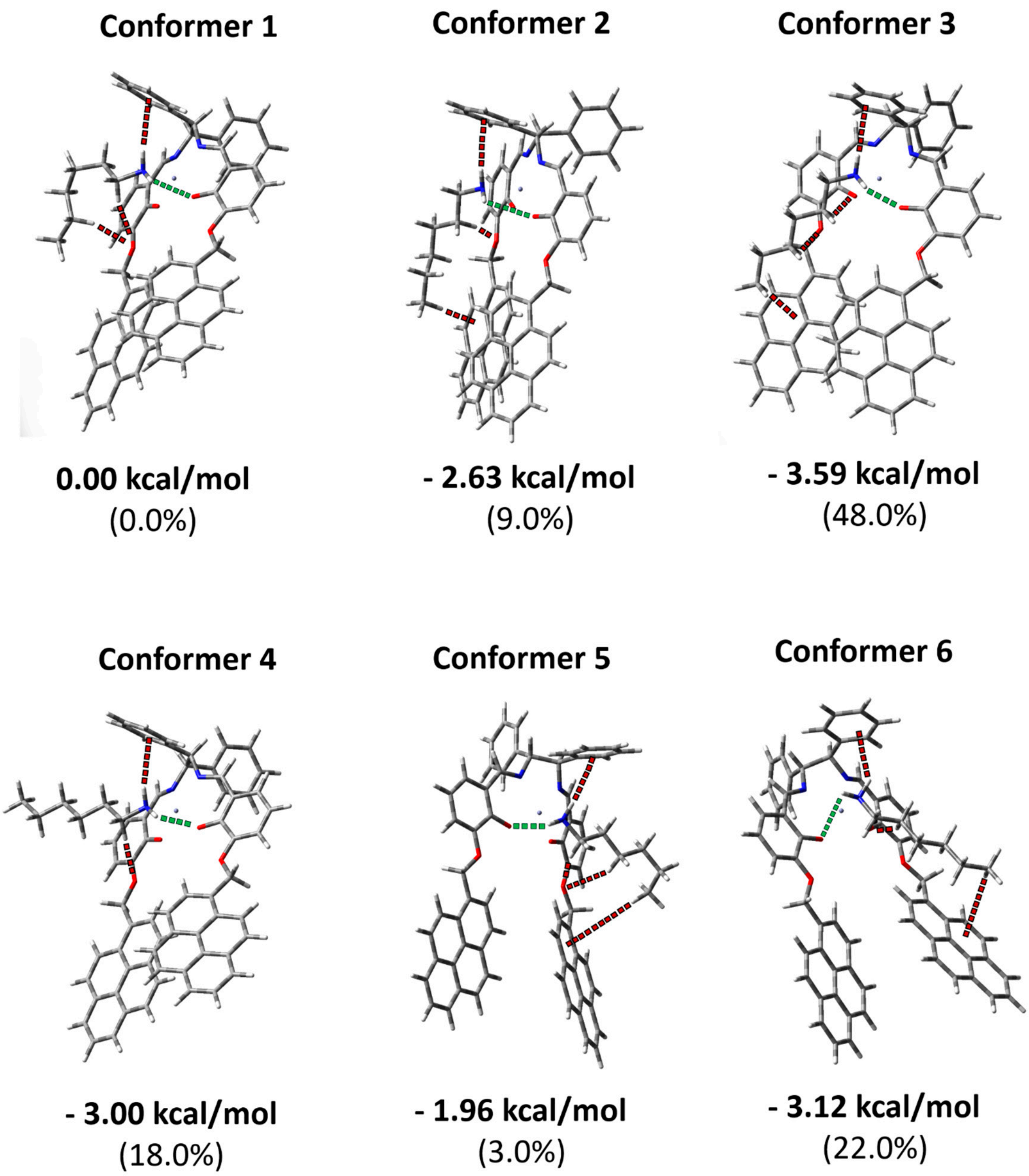

Figure S30. Optimized geometry for the **hexylamine@Zn-Salen-Py** complex in gas phase at B3LYP/6-31G(d,p). NH-O interactions are marked in green while NH-π, CH-π and CH-O interactions in red. Boltzmann distribution is reported between brakets.

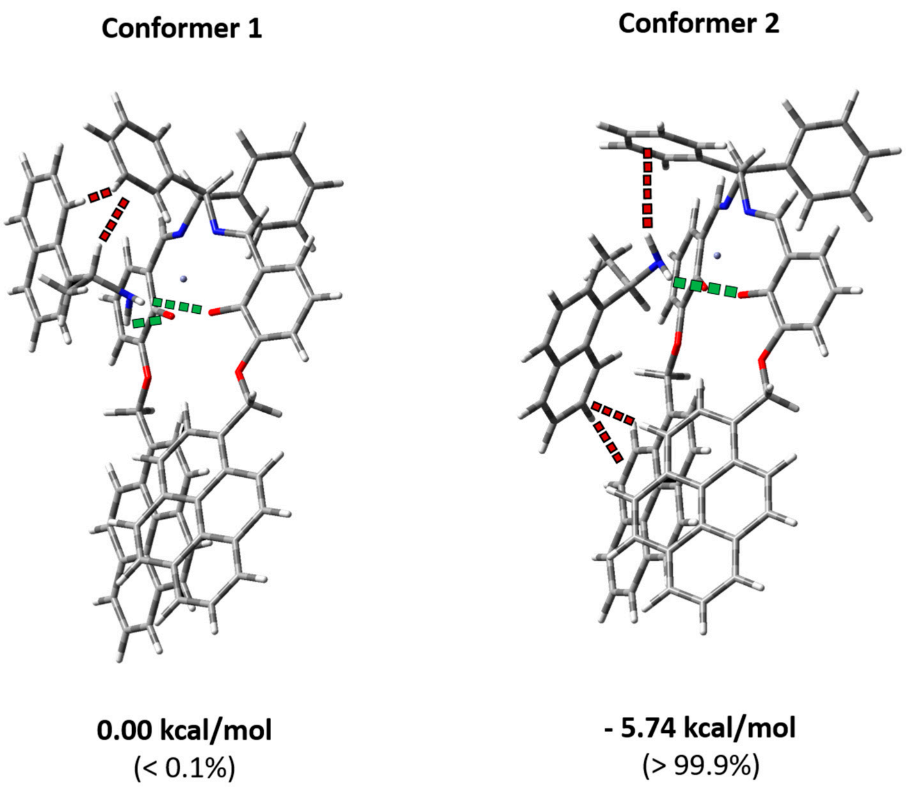

Figure S31. Optimized geometry for the ***R*(+)-1-(2-Naphtyl)ethylamine@Zn-Salen-Py** complex in gas phase at B3LYP/6-31G(d,p). NH-O interactions are marked in green while NH-π, CH-π and CH-O interactions in red. Boltzmann distribution is reported between brakets.

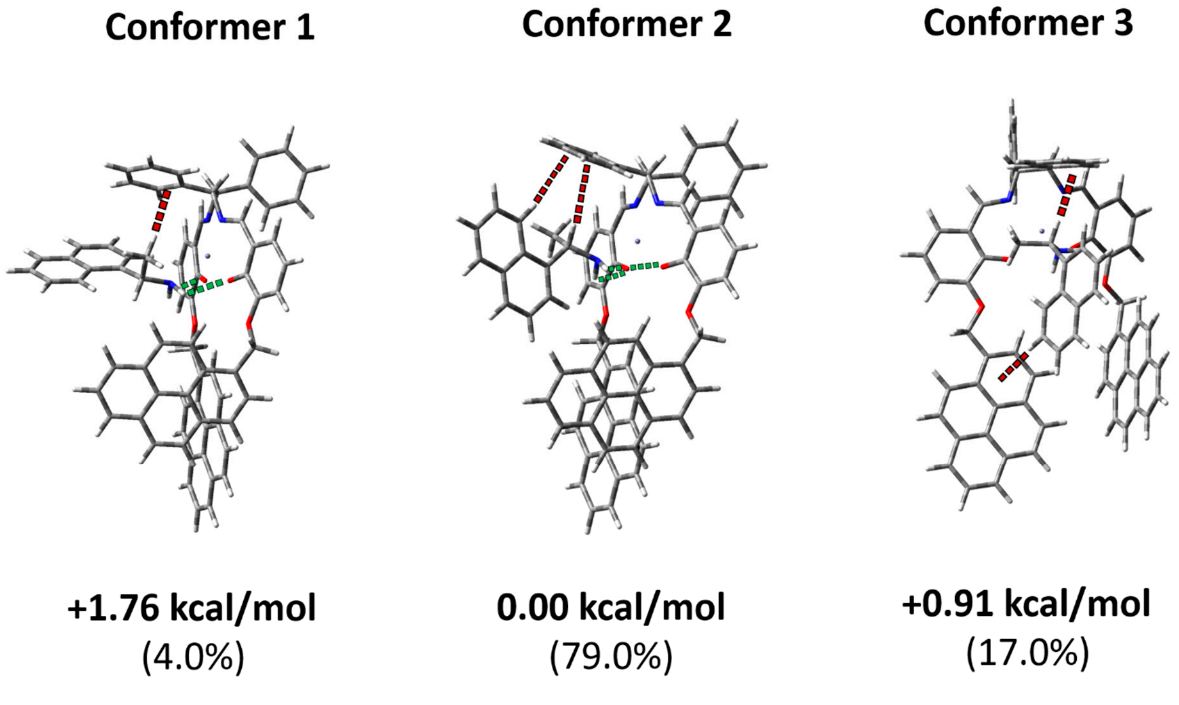

Figure S32. Optimized geometry for the ***S*(-)-1-(2-Naphtyl)ethylamine@Zn-Salen-Py** complex in gas phase at B3LYP/6-31G(d,p). NH-O interactions are marked in green while NH-π, CH-π and CH-O interactions in red. Boltzmann distribution is reported between brakets.
